# A guide to creating design matrices for gene expression experiments

**DOI:** 10.12688/f1000research.27893.1

**Published:** 2020-12-10

**Authors:** Charity W. Law, Kathleen Zeglinski, Xueyi Dong, Monther Alhamdoosh, Gordon K. Smyth, Matthew E. Ritchie

**Affiliations:** 1 The Walter and Eliza Hall Institute of Medical Research, Parkville, 3052, Australia; 2 Department of Medical Biology, The University of Melbourne, Parkville, 3010, Australia; 3 Research and Development, CSL Limited, Bio21 Institute, Parkville, 3010, Australia; 4 School of Mathematics and Statistics, The University of Melbourne, Parkville, 3010, Australia

**Keywords:** Design matrix, model matrix, contrast matrix, statistical models, gene expression analysis

## Abstract

Differential expression analysis of genomic data types, such as RNA-sequencing experiments, use linear models to determine the size and direction of the changes in gene expression. For RNA-sequencing, there are several established software packages for this purpose accompanied with analysis pipelines that are well described. However, there are two crucial steps in the analysis process that can be a stumbling block for many -- the set up an appropriate model via design matrices and the set up of comparisons of interest via contrast matrices. These steps are particularly troublesome because an extensive catalogue for design and contrast matrices does not currently exist. One would usually search for example case studies across different platforms and mix and match the advice from those sources to suit the dataset they have at hand. This article guides the reader through the basics of how to set up design and contrast matrices. We take a practical approach by providing code and graphical representation of each case study, starting with simpler examples (e.g. models with a single explanatory variable) and move onto more complex ones (e.g. interaction models, mixed effects models, higher order time series and cyclical models). Although our work has been written specifically with a **limma**-style pipeline in mind, most of it is also applicable to other software packages for differential expression analysis, and the ideas covered can be adapted to data analysis of other high-throughput technologies. Where appropriate, we explain the interpretation and differences between models to aid readers in their own model choices. Unnecessary jargon and theory is omitted where possible so that our work is accessible to a wide audience of readers, from beginners to those with experience in genomics data analysis.

## Introduction

Gene expression technologies are useful for the study of transcriptomics and their associated profiles amongst biological samples of interest. The technology is used worldwide to examine complex relationships between gene expression (which we will refer to as the *response variable* when performing statistical modelling) and the variables that influence the expression (referred to as *explanatory variables*). From the resulting datasets, careful statistical analysis can be used to find relationships that are of biological interest through the choice of appropriate statistical models applied to the data. The modelling process requires the use of a *design matrix* (or model matrix) that has two roles: 1) it defines the form of the model, or structure of the relationship between genes and explanatory variables, and 2) it is used to store values of the explanatory variable(s) ^1, 2, 3^. Although design matrices are fundamental concepts that are covered in many undergraduate mathematics and statistics courses, their specific and multi-disciplinary application to the analysis of genomic data types through the use of the R programming language adds several layers of complexity, both theoretically and in practice.

This article describes the appropriate design matrix set up for differential expression analyses specific to using the **limma** ^4^ software package, one of the most popular open-source software packages for such analysis worldwide. Our examples have been written for gene expression data, specifically with the assumption that the expression values are genewise log-count per million (log-CPM) measurements from an RNA-sequencing (RNA-seq) experiment. However, most of the concepts and R code covered in this article can also be applied to differential analyses of other genomic data types, including microarrays, ChIP-seq, ATAC-seq, BS-seq, Hi-C and proteomics. The main requirements are that the response data represents abundance on a log-scale and that each row corresponds to an appropriate genomic feature. Typically, the data table from an RNA-seq experiment contains the gene expression measurements for tens of thousands of genes and a small number of samples (usually no more than 10 or 20, although much larger sample sizes are possible). In the modelling process, a single design matrix is defined and then simultaneously applied to each and every gene in the dataset. Rather than demonstrating the application of design matrices across multiple genes, where the modelling concepts are consistent between genes, we simply describe the process for a single gene in our examples. This allows us to illustrate clearly differences between varying models and the implications of adding or removing model parameters.

The article begins by introducing the basic concepts associated with design and contrast matrices. We cover common experimental designs used in genomics research, and move onto more complex study designs as we progress through the sections. We have approached our work from a practical stand-point, with a focus on the R programming inputs and outputs, accompanied by associated plots to illustrate the observed data that we begin with and the fitted models that are produced from a graphical perspective. By omitting most of the theory associated with design matrices, our article allows readers from various backgrounds to gain a better understanding of design matrices, without having statistics as a prerequisite. To enable readers to select the most appropriate design matrix set up for their study design, we also discuss the interpretation of the models and the differences between them.

In each of our examples, we will explicitly display the observed data and include the R code for associated design and contrast matrices that are used in the modelling process. This allows readers to quickly grasp modelling concepts and to apply the R code in their own datasets. Each example is also accompanied by a figure displaying the design matrix and both a written and graphical representation of the statistical model. Whilst the complete data analysis process, from pre-processing data to variance modelling and parameter estimation is not discussed in this article, the design matrices we describe can be implemented in conjunction with the “ *RNA-seq analysis is easy as 1-2-3 with limma, Glimma and edgeR*” differential expression workflow article ^5^ for an RNA-seq analysis beginning with a table of counts.

Other complementary work focusing on design matrices includes that of the CRAN **codingMatrices** package vignette ^6^, which describes the theoretical aspects of design matrices, and the **ExploreModelMatrix** software package ^7^, which allows interactive exploration of design matrices for specified explanatory variables. Although not focusing purely on design matrices, the user’s guides for the **limma** and **edgeR** ^8, 9^ software packages also contain many example case studies for different experimental designs.

## Basic models

### Background

In this section, we outline the general form of some basic models and introduce terminology that will be used in the remainder of the article. The concept of model equations and associated graphical illustrations for fitted models are also introduced here.

### Regression model for covariates

**Figure 1.**
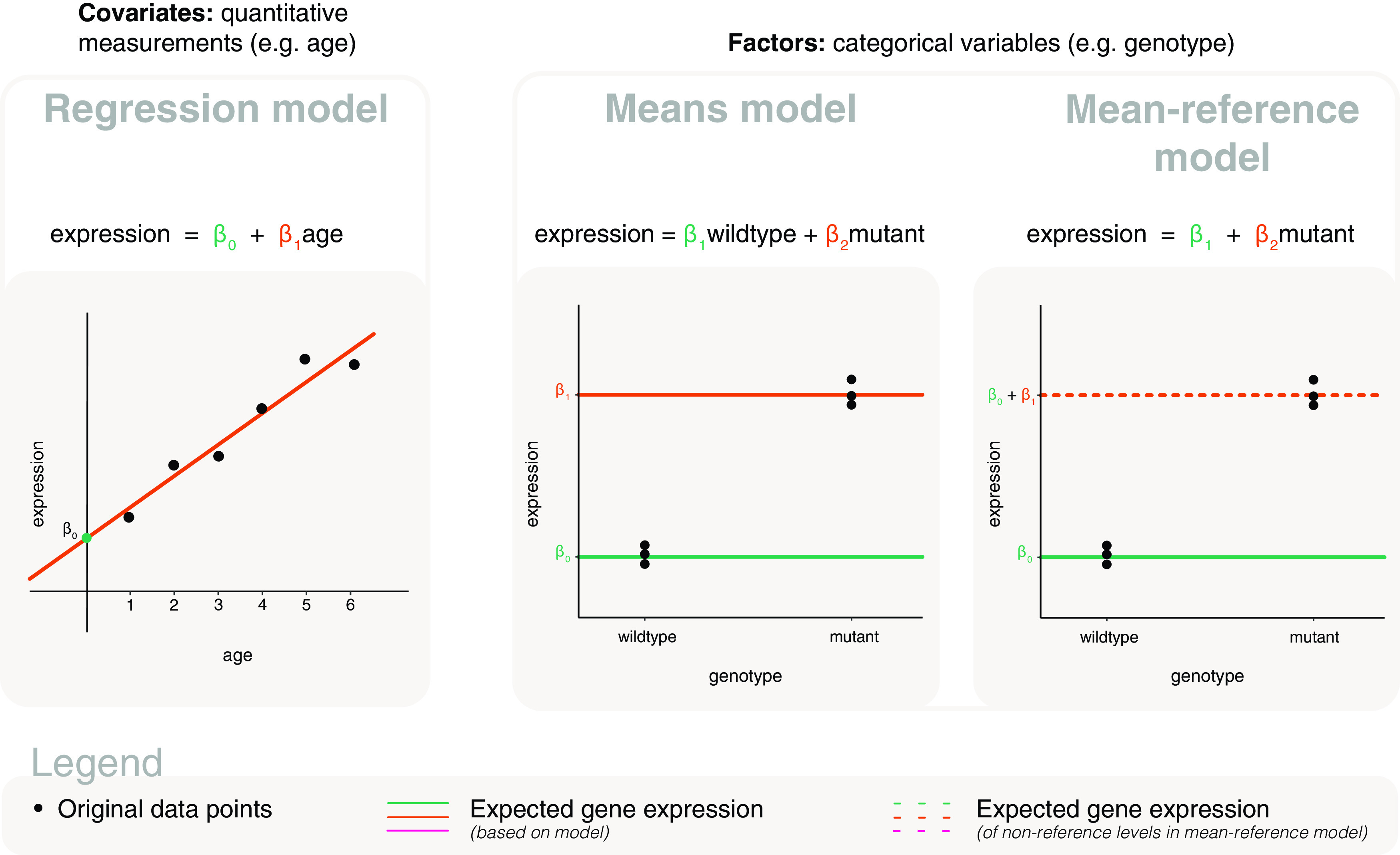
Basic models for covariate and factor explanatory variables. LEFT: The basic model for covariates is referred to as a regression model, which is a line defined by the model parameters *β*_0_ the y-intercept, and *β*_1_ the slope. CENTER: One of two basic models for factors is referred to as a means model, where model parameters are calculated as the mean gene expression of each level of the factor e.g. *β*_1_ represents the mean expression for wildtype and *β*_2_ represents the mean of mutant. RIGHT: The other basic model we refer to for factors is a mean-reference model, where the first model parameter is calculated as the mean gene expression of the reference level, and subsequent parameters are calculated relative to the reference level e.g. *β*_1_ represents the mean expression for wildtype and *β*_2_ represents the difference between mutant and wildtype. In each plot, the points represent the original data; coloured lines are used to represent expected gene expression, where dashed lines are specifically used to represent expected gene expression for non-reference levels in the mean-reference model e.g. mutant.

To begin with, let us consider two types of explanatory variables: *covariates* and *factors*. Covariates contain numerical values that are quantitative measurements associated with samples in the experiment. These can be the age or weight of an individual, or other molecular or cellular phenotypes on a sample, such as measurements obtained from a polymerase chain reaction (PCR) experiment or fluorescence activated cell sorting (FACS). For covariates, it is generally of interest to know the rate of change between the response and the covariate, such as “how much does the expression of a particular gene increase/decrease per unit increase in age?”. We can use a straight line to model, or describe, this relationship, which takes the form of

$$\text{expression}=\beta_{0}+\beta_{1}\text{age}$$

where the line is defined by *β*_0_ the y-intercept and *β*_1_ the slope ( Figure 1). In this model, the age covariate takes continuous, numerical values such as 0.8, 1.3, 2.0, 5.6, and so on. We refer to this model generally as a *regression model*, where the slope indicates the rate of change, or how much gene expression is expected to increase/decrease by per unit increase of the covariate. The y-intercept and slope of the line, or the *β*s ( *β*_0_ and *β*_1_), are referred to as the model *parameters*. The true values of the parameters are unknown, but are estimated in the modelling process. A positive estimate of a model parameter indicates that an explanatory variable has a positive influence on gene expression (an increasing slope), whilst a negative value indicates that the explanatory variable has a negative influence on gene expression (a decreasing slope). In some cases, one may convert the age covariate into a factor by categorising the smaller values as “young” and larger values as “mature”, and instead use the models described below.

### Means model for factors

Factors are categorical variables or classifiers associated with samples in the experiment. They are often separated into those that are of a biological nature (e.g. disease status, genotype, treatment, cell-type) and those that are of a technical nature (e.g. experiment time, sample batch, handling technician, sequencing lane). Unique values within a factor are referred to as *levels*. For example, genotype as a factor may contain two levels, “wildtype” and “mutant”. Here, it is generally of interest to determine the expected or mean gene expression for each state or level of the factor. The relationship between gene expression and the factor can be described, or modelled, in the form of

$$\text{expression}=\beta_{1}\text{wildtype}+\beta_{2}\text{mutant}$$

where *β*_1_ represents the mean gene expression for wildtype, and *β*_2_ represents the mean gene expression for mutant ( Figure 1). Unlike that of the age covariate which can take on any non-negative numerical value in the model, the levels of genotype can only take on the values of 0 or 1. For example, wildtype is equal to 1 (and mutant is equal to 0) when determining the expected expression for the wildtype group, such that

$$\text{expression}=\beta_{1}\;\text{for wildtype.}$$

Similarly, mutant is equal to 1 (and wildtype is equal to 0) when determining the expected expression for the mutant group, such that

$$\text{expression}=\beta_{2}\;\text{for mutant.}$$

Notice that wildtype and mutant “take turns” in taking on the 0 and 1 values. This is because the categorisation of samples as wildtype or mutant are mutually exclusive, where a sample cannot be both wildtype and mutant, or neither wildtype nor mutant. The model estimates expected gene expression as *β*_1_ or *β*_2_, where *β*_1_ is calculated as the mean of observed expression values in wildtype, and *β*_2_ is calculated as the mean of observed expression values in mutant. In other words, the *β*s (or model parameters) are estimated as the mean of each level in the genotype factor, as depicted in Figure 1 as distinct solid lines.

Each of the horizontal lines in Figure 1 are defined by their y-intercept (and a slope of 0), and are themselves regression models. We, however, will refer specifically to models of this type as a *means model* since the model parameters represent the group means. This also allows us to differentiate these models from the general regression models applied to covariates where the y-intercept and slope can both be non-zero. As noted for covariates, the true values for the model parameters are unknown but estimable. Whilst the expected expression of each factor level is informative, it is often the difference in expression between levels that are of key interest, e.g. “what is the difference in expression between wildtype and mutant?”. These differences are calculated using linear combinations of the parameters (a fancy way to say that we multiply each parameter by a constant) which we refer to as *contrasts*. For example, a contrast of (1,−1) calculates *β*_1_ − *β*_2_, the difference in means between wildtype and mutant.

### Mean-reference model for factors

An alternative parameterisation of the means model directly calculates the gene expression difference between mutant and wildtype. It does so by using one of the levels as a reference. Such a model is parameterised for the mean expression of the reference level (e.g. wildtype), and the rest of the levels are parameterised relative to the reference (e.g. the difference between mutant and wildtype). The relationship between gene expression and genotype is modelled in the form of

$$\text{expression}=\beta_{1}+\beta_{2}\text{mutant}$$

where *β*_1_ represents the mean gene expression for wildtype, and *β*_2_ is the difference between means of mutant and wildtype ( Figure 1). Here, mutant in the equation takes the value of 0 when determining the expected expression for the wildtype group, such that

$$\text{expression}=\beta_{1}\;\text{for wildtype.}$$

On the other hand, mutant is equal to 1 when determining the expected expression for the mutant group, such that

$$\text{expression}=\beta_{1}+\beta_{2}\;\text{for mutant.}$$

Expected gene expression for wildtype is represented directly by the first model parameter, *β*_1_, and depicted as a solid line in Figure 1. Whilst the expected gene expression for mutant is calculated as the sum of both parameters, and represented by a dashed line in in Figure 1. Like the means model, the model demonstrated here is a regression model in itself. We, however, refer to this model specifically as a *mean-reference model* to distinguish it from the general model form that we use for covariate explanatory variables. The means model and the mean-reference model are equivalent models that differ in parameterisation, such that the form of the model is different but one could obtain equivalent values for the expected gene expression of wildtype and mutant from both models.

### Terminology

The terminology and concepts covered in this section are summarised in the table below, in the context of modelling gene expression data. The table also extends to some definitions and descriptions covered later in the article, and is a useful resource to refer to from time-to-time.

**Table:** 

Term	Description
Response variable	Gene expression, e.g. log-CPM values.
Explanatory variable	Variable that influences gene expression.
Statistical model	Used to describe the relationship between response and explanatory variables.
Model parameters	Of statistical models, unknown but estimable values that describe the direction and magnitude with which explanatory variables influence gene expression.
Design matrix	Used to define the form of a statistical model and to store observed values of the explanatory variable(s). Used in the computation process to estimate model parameters.
Contrast matrix	Used in conjunction with a design matrix to calculate specific values of interest between estimated parameters.
Covariate	Explanatory variable that is numerical in nature, e.g. age.
Y-intercept	Point at which a model prediction crosses the y-axis.
Slope	Rate of change for a model e.g. the change in gene expression per unit increase of a covariate.
Regression model	Our reference to statistical models for covariates.
Factor	Explanatory variable that is categorical in nature, e.g. genotype.
Levels	Unique values within a factor, e.g. wildtype or mutant.
Means model	Our reference to statistical models for factors where parameters are calculated as the mean of each factor level.
Contrasts	Linear combinations of estimated parameters. A contrast matrix is made up of individual contrasts.
Mean-reference model	Our reference to statistical models for factors where parameters are calculated as the mean reference level, and relative means for subsequent levels.
Fitted model	The statistical model written with estimates of the model parameters. In our figures, we draw the fitted model (expected gene expression) along with the data points (observed gene expression) to give an idea of how well the fitted model represents the relationship between response and explanatory variables.
Additive effect	When the combined effect of two factors equals the sum of the two individual effects, e.g. if the estimated effect of Group A is *κ* and the estimated effect of sequencing on lane I is *τ*, then a sample in Group A that is sequenced on lane I has an expected expression of *κ* + *τ* if the two factors have an additive effect.
Interaction effect	When the combined effect of two factors does not equal the sum of the two individual effects, e.g. for the example above, a sample in Group A that is sequenced on lane I has an expected expression of *κ* + *τ* + *δ* if the two factors have an interaction effect, where *δ* can be a positive or negative number.
Nested factors	A factor is considered to be nested within a second factor, e.g. group is nested within batch, if different sets of its levels can be found in each level of the second factor, e.g. group A and group B are processed in batch B1 and group C and group D are processed in batch B2.
Mixed effects models	A statistical model that contains both fixed and random effects, where random effects are usually not of interest to the study at hand.

## Overview of models fitted

In the sections to follow, we explore various models for explanatory variables that are factors, starting from the most basic study designs to those that are more complex. We then cover some models for explanatory variables that are covariates. The tables below summarise the data examples, R input for the associated design matrices, and the sections from which they can be found. In the tables, factors are distinguished from covariates by the presence of subscripts listing their levels e.g. “factor _*LEVEL*1, *LEVEL*2_”. Associated sections are marked with an asterisk if the design matrix cannot be sufficiently summarised within the table.

### Design and contrast matrices

This section describes and compares models that are coded with and without an intercept term for covariates and factors. It also shows the fundamental elements for computing differences between model parameters using contrasts and contrast matrices.

**Table:** 

Explanatory variables	Design matrix	Section
age	model.matrix(∼age) model.matrix(∼0+age)	Covariates: With intercept Covariates:Without intercept
group _*HEALTHY*, *SICK*_	model.matrix(∼group) model.matrix(∼0+group)	Factors: With intercept Factors: Without intercept

### Study of treatments and control

This section examines a study on four treatment groups (CTL, I, II, and III). The example here represents a study design that is very common in practice, where there are several treatments (or conditions or groups) including a control. Comparisons between the levels are computed using two alternative design matrices. In this section, we also look at more complex set ups for contrasts to compute comparisons that may be of interest.

**Table:** 

Explanatory variables	Design matrix	Section
treatment _*CTL*, *I*, *II*, *III*_	model.matrix(∼treatment) model.matrix(∼0+treatment)	Treatment versus control All pairwise comparisons

### Study of interactions and additivity of treatments

In this section we consider the effect of combining two separate treatments. Our first example looks at the interactivity of the treatments using a model from the previous section, which has a single treatment factor. We then show an alternative method to calculate the same estimates using a two factor model.

**Table:** 

Explanatory variables	Design matrix	Section
treat1 _*NO*, *YES*_ and treat2 _*NO*, *YES*_	model.matrix(∼treat1*treat2) model.matrix(∼treat1+treat2)	Interactions using a two-factor model Additivity using a two-factor model

### Studies with multiple factors

This section looks at studies with multiple factors. It includes study designs that are more complex in nature and describes the approaches one can take to examine the differences of interest. The section covers studies with nested factors and the fitting of mixed effects models.

**Table:** 

Explanatory variables	Design matrix	Section
tissue _*LUNG*, *BRAIN*_ and cells _*B*, *T*_	model.matrix(∼0+group) *with* ${\text{group}}_{LUNG\_B,BRAIN\_B,LUNG\_T,BRAIN\_T}$	Conversion to a single factor
group _*A*, *B*_, lane _*L*1, *L*2_ and technician _*I*, *II*_	model.matrix(∼0+group+lane+technician)	Accounting for factors that are not of interest
group _*A*, *B*_ and batch _*B*1, *B*2_	*Check rank of design matrix*	Nested factors and matrices without full rank*
treatment _*X*, *Y*_ and timepoint _*T*1, *T*2_ with repeated measurements	*Model mouse IDs, then add columns representing timepoint T2 for both treatments*	Time series experiment with repeated mouse measurements nested within treatments*
treatment _*X*, *Y*_ and timepoint _*T*1, *T*2_ with repeated measurements	model.matrix(∼0+group) *with* ${\text{group}}_{X\_T1,X\_T2,Y\_T1,Y\_T2}$, *and mouse ID as random effect*	Treating factors that are not of direct interest as random effects*

### Studies with covariates

In this section, we look at explanatory variables that are covariates rather than factors. We begin with fitting some simple models and work up towards more complex ones such as the fitting of cyclical models.

**Table:** 

Explanatory variables	Design matrix	Section
treatment _*X*, *Y*_ and time	model.matrix(∼0+treatment*time)	Combination with factor variable
time	model.matrix(∼time)	Linear time series
time	model.matrix(∼poly(time, degree=2, raw=TRUE))	Quadratic time series
time	model.matrix(∼poly(time, degree=3, raw=TRUE))	Cubic time series
time	model.matrix(∼time+sinphase+cosphase) model.matrix(∼sinphase+cosphase)	Cyclical models* Cyclical models*

## Design and contrast matrices

### Background

In this section, we demonstrate how design and contrast matrices can be created for the most basic experimental designs with covariates and factors. Specifically, we discuss the similarities and differences between design matrices that include and exclude an intercept term.

**Figure 2.**
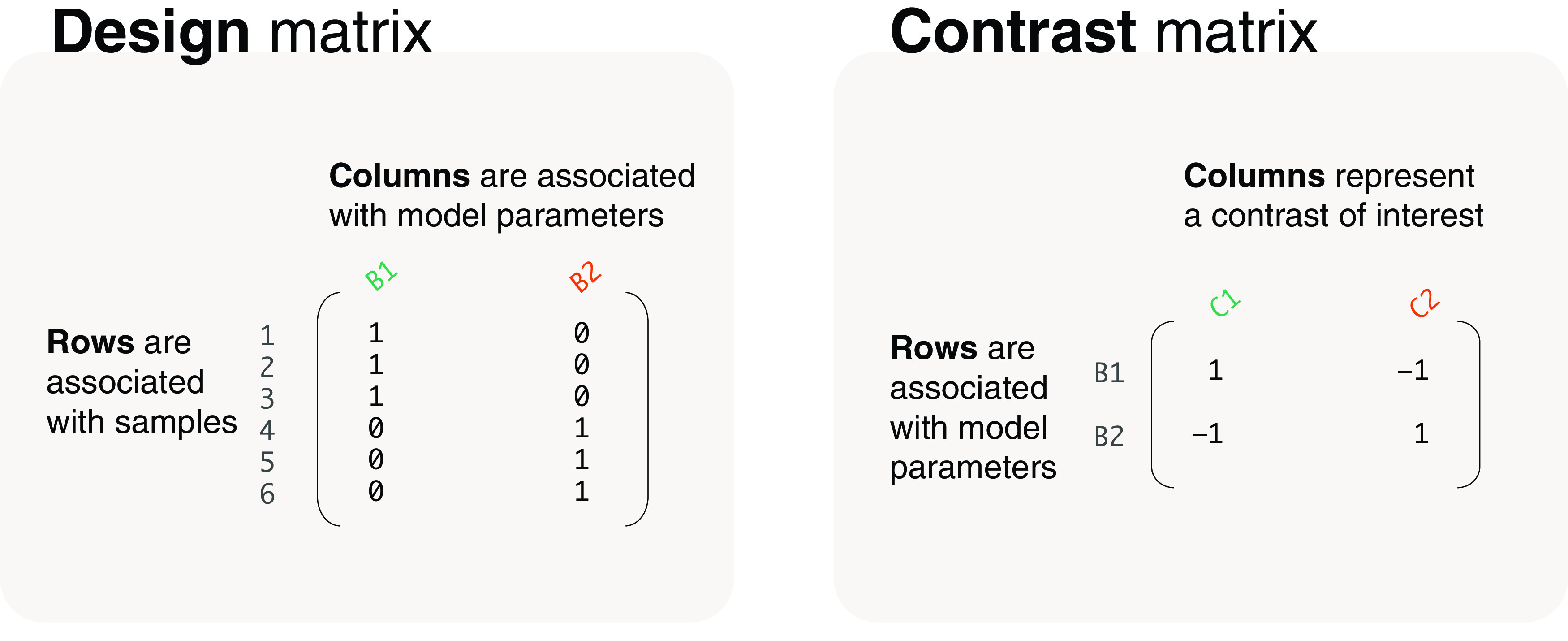
The structure of design and contrast matrices.

Design matrices are used in the estimation process of model parameters. The design matrix has columns associated with the parameters and rows associated with samples ( Figure 2). If the estimated parameters are not of direct interest, a *contrast matrix* can be used to calculate contrasts of the parameters. Combining multiple contrasts, each column in the contrast matrix represents a single contrast, and has rows associated with columns in the corresponding design matrix ( Figure 2). Using the R programming language, we code for design matrices using the model.matrix function from the **stats** package, and contrast matrices using the makeContrasts function from the **limma** package. To learn more about these functions, one can bring up associated help pages by typing ?stats::model.matrix and ?limma::makeContrasts in the R console.

### Design matrices with and without intercept term

For a single explanatory variable, which we simply call variable, a design matrix can be coded by model.matrix(~variable) to include an intercept term, or by model.matrix(~0+variable) to exclude the intercept term. One of the most fundamental concepts in the coding of design matrices is to understand when one should include an intercept term, when not to, and how it affects the underlying model. If variable is a factor, then the two models with and without the intercept term are equivalent, but if variable is a covariate the then two models are fundamentally different.

### Covariates

Using age as an example, let’s look at the gene expression of mice where their age in weeks from birth were recorded. The expression of a single gene is recorded as a numerical vector called expression. The age of mice is also recorded as a numerical vector in age (as weeks), and we use an additional mouse character vector to show that these are independent measurements. The three vectors, expression, mouse and age, that represent our example data are displayed below as a data frame as follows:



##   expression  mouse age
## 1       2.38 MOUSE1   1
## 2       2.85 MOUSE2   2
## 3       3.60 MOUSE3   3
## 4       4.06 MOUSE4   4
## 5       4.61 MOUSE5   5
## 6       5.04 MOUSE6   6



#### With intercept

**Figure 3.**
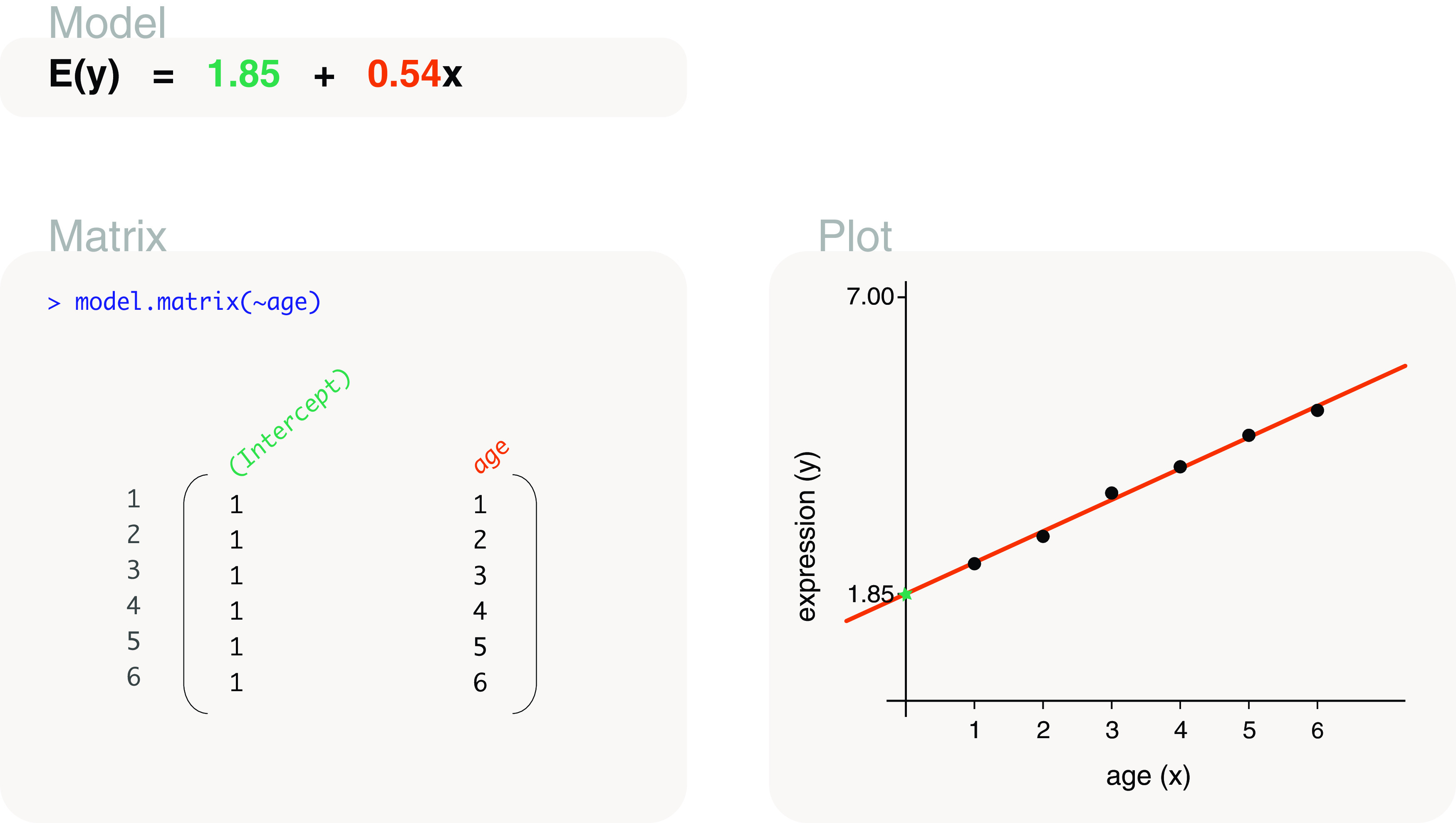
Expected gene expression is modelled by an age covariate, which is denoted as *x* in the model and plot. This particular model includes an intercept term so that the model (line) has flexibility in intersecting the y-axis at any point. MODEL: The fitted model in written form, where *y* represents gene expression and *E*( *y*) expected gene expression. Estimated model parameters are highlighted in colour. MATRIX: R input and output for the associated design matrix, with the colour of column names (model parameters) matching that of the estimated parameters above. For simplicity, ”assign” and ”contrasts” attributes of the design matrix are not displayed in our figures (see ‘?stats::model.matrix’ for more). PLOT: Observed data points are drawn together with the fitted model representing expected gene expression. Where appropriate, aspects of the fitted model are drawn in a colour that matches associated parameter estimates.

A design matrix with an intercept term can be coded as model.matrix(~age). The resultant design matrix, which is displayed in Figure 3, contains a column of 1s (the intercept term) and a column with values taken from age. This design matrix is associated with a regression model (see Figure 1), where the intercept term in the first column is parameterised for the y-intercept, and “age” in the second column is parameterised for the slope of the regression line. In other words, the second column is used to estimate the rate of change in gene expression per unit increase in age.

The parameters can be estimated as 1.85 for the y-intercept and 0.54 for the slope. This means that for every 1 unit (or week) increase in age, gene expression increases by a value of 0.54 on average. We can write our statistical model using the estimated model parameters to give us our *fitted model*. The fitted model can be written as *E*( *y*) = 1.85 + 0.54 *x*, with *y* representing expression of the gene, *E*( *y*) representing the expected gene expression and *x* representing age.

#### Without intercept

**Figure 4.**
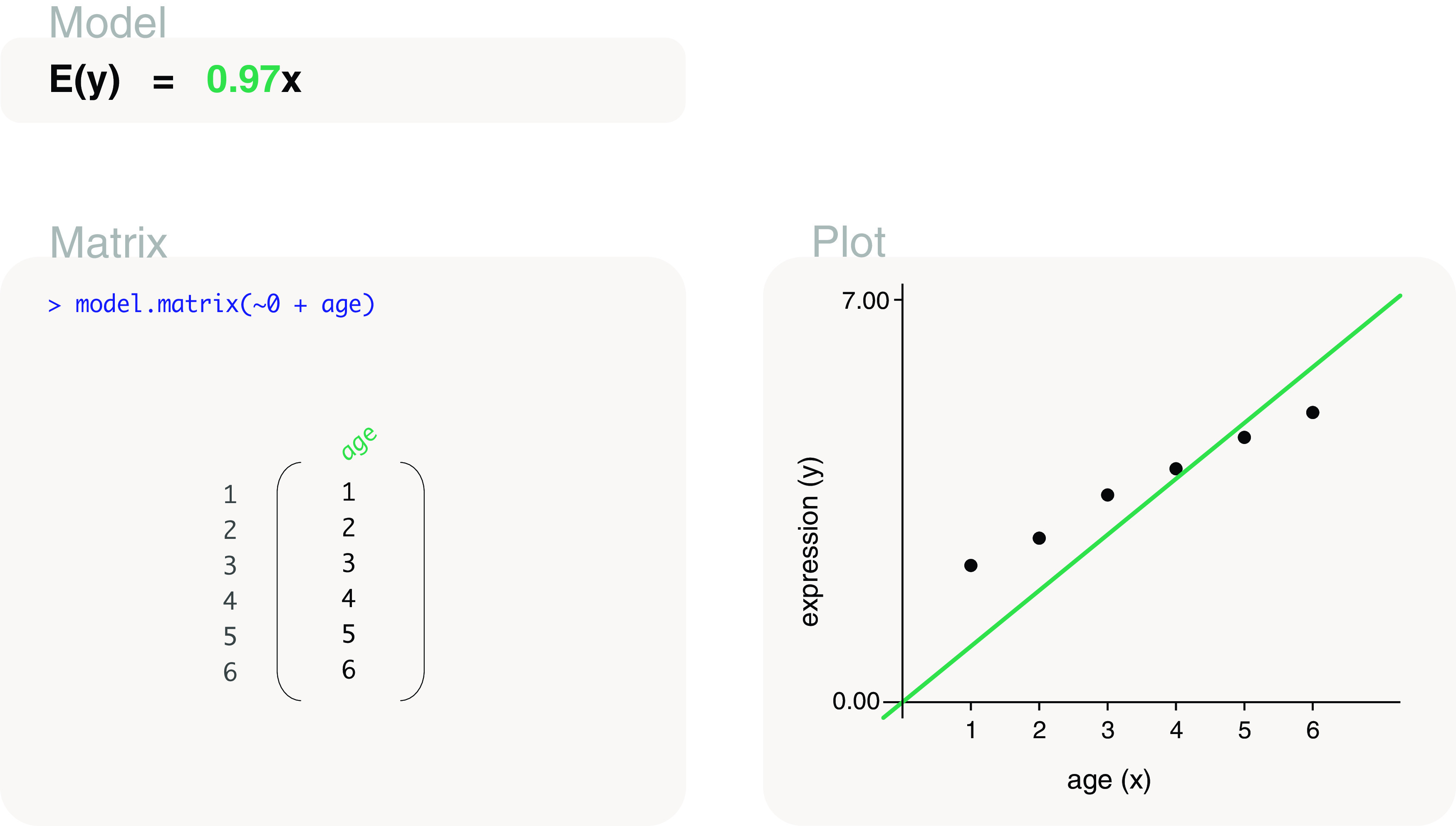
Expected gene expression modelled by an age covariate, where the model (line) must intersect the y-axis at the zero-point. This restriction is due to the design matrix set up which excludes the intercept term.

Alternatively, a design matrix without an intercept term can be coded as model.matrix(~0+age). This design matrix contains a single column that represents age, as shown in Figure 4, which is parameterised for the slope of a regression line. By adding a 0 to the formula in model.matrix, the intercept term has been removed. This means that the regression line is forced to intercept the y-axis at 0. The slope of the line can be estimated to be 0.97, such that gene expression is expected to increase by 0.97 for every 1 unit increase in age. The fitted model is written as *E*( *y*) = 0.97 *x*.

#### Comparing models

When comparing between the models with and without an intercept term, we observe that the model without an intercept term ( Figure 4) does not fit as closely to the observed data as the model with an intercept term ( Figure 3). It is not surprising that the model with an intercept term provides a better fit to the data since it is less restrictive (allows the y-intercept to be at any point) than the model without an intercept term. The extra parameter in the model allows it to be more flexible. In general, we suggest the inclusion of an intercept term for modelling explanatory variables that are covariates since it provides a more flexible fit to the data points. A model without an intercept term would only be recommended in cases where there is a strong biological reason why a zero covariate should be associated with a zero expression value, and such contexts are rare in gene expression modelling.

### Factors

**Figure 5.**
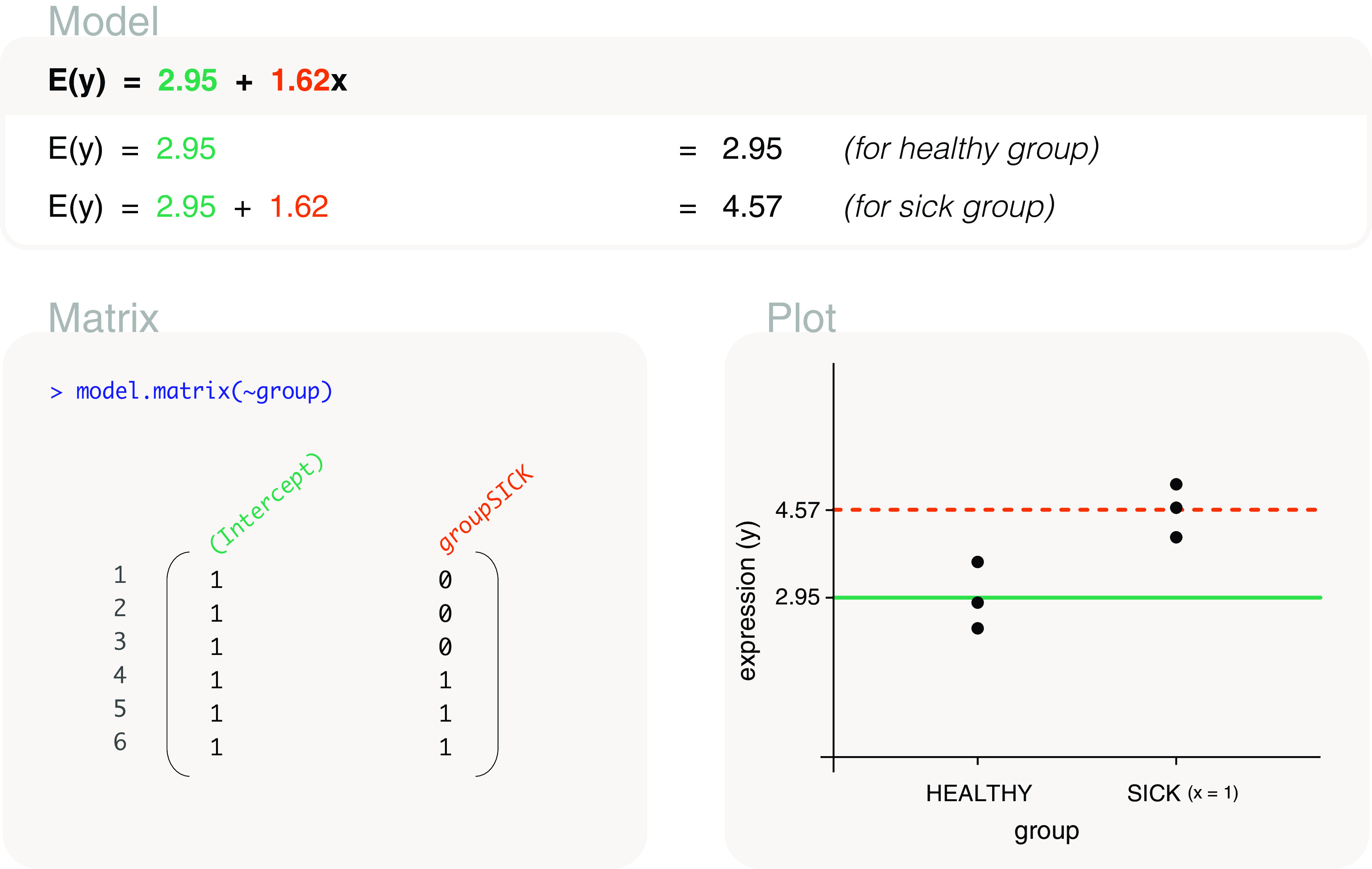
Expected gene expression is modelled by a group factor, where *x* is an indicator variable for sick mice ( *x* = 1 for sick mice, and *x* = 0 otherwise). The associated design matrix includes an intercept term, where healthy mice acts as the reference level. The expected gene expression of non-reference levels, e.g. that of sick mice, are represented by dashed lines in the plot.

**Figure 6.**
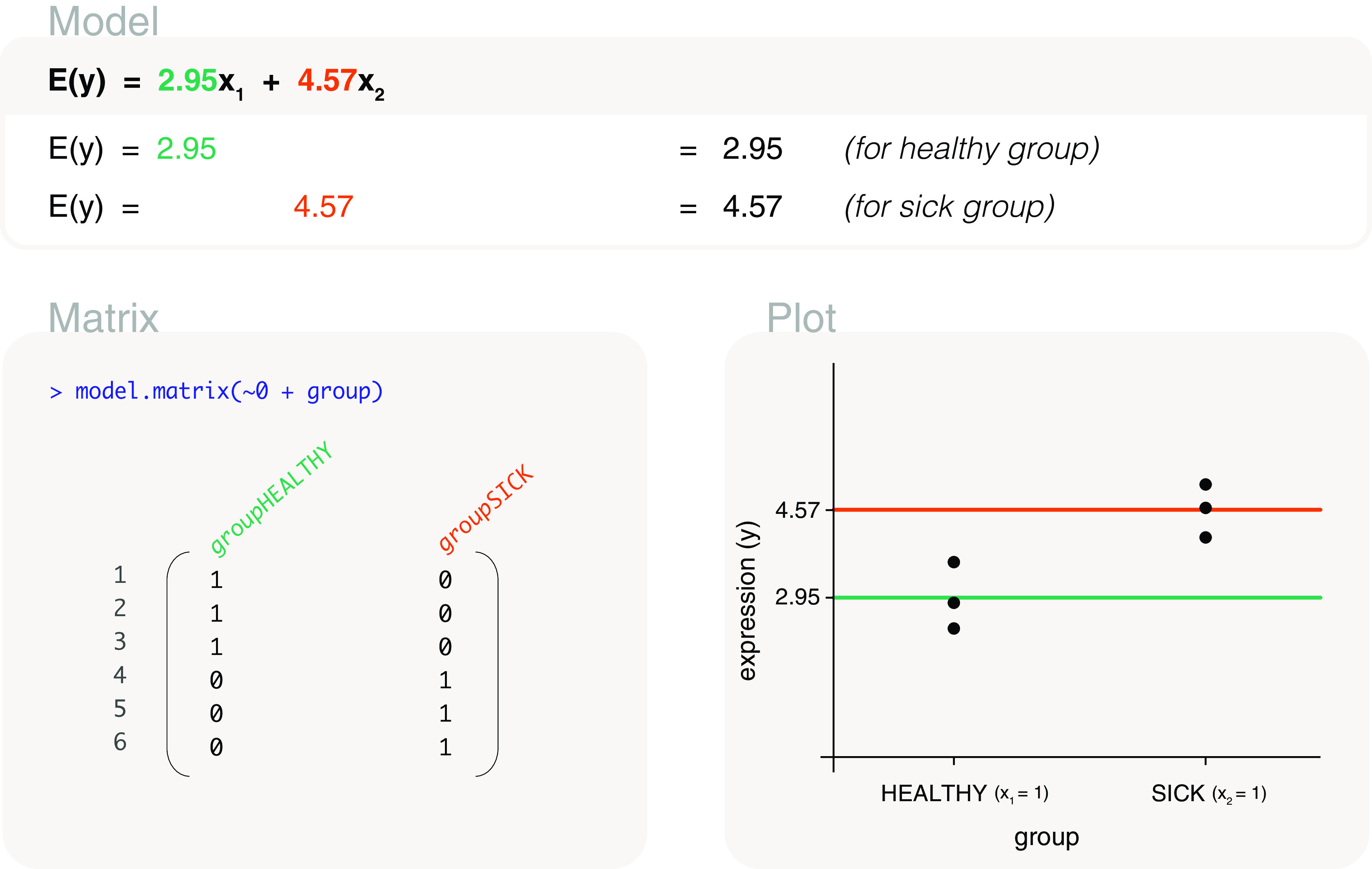
Expected gene expression is modelled by a group factor, where *x*_1_ is an indicator variable for healthy mice ( *x*_1_ = 1 for healthy; 0 otherwise), and *x*_2_ is an indicator variable for sick mice ( *x*_2_ = 1 for sick; 0 otherwise). The associated design matrix excludes an intercept term.

Now we consider an example of gene expression on healthy and sick mice, each in triplicate. Healthy and sick mice are classified using a group factor which contains two levels, HEALTHY and SICK. The level names are written in all capitals so that design matrices have column names that are easier to read by default e.g. “groupSICK” ( Figure 5 and 6) rather than “groupsick” or “groupSick”. The data is displayed by combining vectors for expression, mouse and group as follows:



##   expression  mouse   group
## 1       2.38 MOUSE1 HEALTHY
## 2       2.85 MOUSE2 HEALTHY
## 3       3.60 MOUSE3 HEALTHY
## 4       4.06 MOUSE4    SICK
## 5       4.61 MOUSE5    SICK
## 6       5.04 MOUSE6    SICK



#### With intercept

A design matrix with an intercept term can be coded as model.matrix(~group) to obtain a two column matrix ( Figure 5). In general, the resulting design matrix will have the same number of columns as the factor group has levels. The design matrix contains a column of 1s (the intercept term), and a column with values as 0s or 1s (a value of 1 when the associated sample is in the sick group, and 0 otherwise). This design matrix is parameterised for a mean-reference model ( Figure 1), where the intercept term in the first column is parameterised for the mean expression of the healthy group, and the second column is parameterised for the mean expression of the sick group relative to healthy (difference between sick and healthy). The healthy group is selected as the reference level since HEALTHY is the first level in group. Levels in a factor are ordered alphanumerically by default, but re-specification of the reference can be carried out using the relevel function in the **stats** package. Using this design matrix, the parameters can be estimated to be 2.95 and 1.62, such that the mean gene expression of the healthy group is 2.95, and the mean gene expression of the sick group relative to healthy is 1.62. We can calculate the mean expression of the sick group by summing both parameter estimates, which in this case is equal to 4.57. Using the estimated values, the fitted model for expected gene expression can be written as *E*( *y*) = 2.95 + 1.62 *x*, where *x* is an indicator variable for mice that are sick. The indicator variable, or *x*, takes the value of 1 when calculating the expected expression of sick mice, and takes the value of 0 when calculating the expected expression of healthy mice. In other words, *E*( *y*) = 2.95 for healthy mice and *E*( *y*) = 4.57 for sick mice.

#### Without intercept

A design matrix without an intercept term can be coded as model.matrix(~0+group), which gives an equivalent model to that of the previous model. The design matrix here also contains two columns ( Figure 6), but is instead parameterised for a means model ( Figure 1). This means that the first column of the design matrix is parameterised for the mean expression of the healthy group, where a value of 1 is present when the associated sample belongs to the healthy group, and 0 otherwise. The second column is parameterised for the mean expression of the sick group, and has a value of 1 when the associated sample belongs to the sick group, and 0 otherwise. The parameters in this model can be estimated as 2.95 and 4.57 for the mean gene expression of healthy and sick mice, respectively. Thus, the fitted model for expected gene expression can be written as *E*( *y*) = 2.95 *x*_1_ + 4.57 *x*_2_, where *x*_1_ and *x*_2_ are indicator variables for healthy mice and sick mice respectively. In other words, *E*( *y*) = 2.95 for healthy mice and *E*( *y*) = 4.57 for sick mice.

#### Comparing models

As mentioned in our earlier description of basic models ( Figure 1), models with and without an intercept term are equivalent for factor explanatory variables, but differ in parameterisation. This means that the expected expression values for healthy and sick mice are the same regardless of whether a means model (without an intercept term in the design matrix) or a mean-reference model (with an intercept term in the design matrix) is fitted. The only difference is that the expected gene expression for sick mice is calculated by summing both parameter estimates in a mean-reference model, whereas it is estimated directly as the second parameter in a means model. For this reason, it ultimately does not matter which design matrix is used. We recommend the use of whichever design matrix that is better understood by the reader, which is often the design matrix without the intercept term since the interpretation of parameters is more straightforward.

### Contrast matrix for computing differences

When fitting a means model, the parameter estimates themselves are usually not of direct interest. It is the difference between the parameter estimates, or difference between mean expression of groups, that is of interest. The difference in parameter estimates can be calculated using a contrast matrix via the makeContrast function. To specify the comparison of interest, column names from the design matrix, “groupHEALTHY” and “groupSICK”, are inserted into the function. The design matrix and associated contrast matrix is coded as follows:



design <- model.matrix(~0+group)
makeContrasts(groupSICK-groupHEALTHY, levels=colnames(design))

##               Contrasts
## Levels         groupSICK - groupHEALTHY
##   groupHEALTHY                       -1
##   groupSICK                           1



The makeContrast function simply creates the contrast of (-1, 1) which subtracts the first parameter estimate (mean expression of healthy) from the second parameter estimate (mean expression of sick). Using the parameter estimates estimated earlier ( Figure 6), the contrast calculates -2.95 plus +4.57 which equals 1.62. In other words, we expect gene expression of sick mice to be upregulated by 1.62 units relative to healthy mice. Notice how this is the same value as the second parameter estimate in the mean-reference model ( Figure 5), since that model is directly parameterised for the difference between sick and healthy mice. It is also reasonable to compute the difference in the opposite direction, by having groupHEALTHY-groupSICK as the first argument of the makeContrasts function. This will result in the value of -1.62 instead. The two options only differ in their interpretation, “gene expression of sick mice is greater than healthy mice by a value of 1.62” versus “gene expression of healthy mice is greater than sick mice by a value of -1.62”.

## Study of treatments and control

### Background

In this section, we focus on a single factor as an explanatory variable to modelling gene expression. The factor we use contains several levels, which allows us to discuss some common comparisons of interest, and show different methods of calculating those differences.

A very common study design examines several conditions of interest, where one condition represents the control. Considering such an experimental design, we want to model the relationship between gene expression and four possible conditions: three treatments and a control. The explanatory variable is set up as a factor vector, which we have named treatment, and the factor is used to classify samples into the control group (CTL), treatment I, treatment II, and treatment III. The factor has a total of 4 levels. Gene expression is recorded as a numeric vector called expression, and mouse is a character vector showing that the observations are independent measurements. The data combines the vectors as follows:



##    expression   mouse treatment
## 1        1.01  MOUSE1       CTL
## 2        1.04  MOUSE2       CTL
## 3        1.04  MOUSE3       CTL
## 4        1.99  MOUSE4         I
## 5        2.36  MOUSE5         I
## 6        2.00  MOUSE6         I
## 7        2.89  MOUSE7        II
## 8        3.12  MOUSE8        II
## 9        2.98  MOUSE9        II
## 10       5.00 MOUSE10       III
## 11       4.92 MOUSE11       III
## 12       4.78 MOUSE12       III



We know from the previous section that the treatment factor can be represented in a means model or a mean-reference model using the design matrices coded as model.matrix(~0+treatment) or model.matrix(~treatment) respectively. Either representation would give equivalent models, and so it would be unnecessary to describe both models for the same exercise. Based on the comparison of interest at hand, we demonstrate the use of one of the models using the most direct approach.

### Treatments versus control

For a comparison of each treatment group versus the control group, we model gene expression using a mean-reference model. This is ideal since the differences can be estimated directly from the model parameters, and without the use of an additional contrast matrix. To do this, the control group would act as the reference and must be the first level of the treatment factor vector. We can view the order of levels by levels(treatment). If the level associated with the control group is not listed first, it can be changed to the first or reference level with the code treatment <- relevel(treatment, ref="CTL").

**Figure 7.**
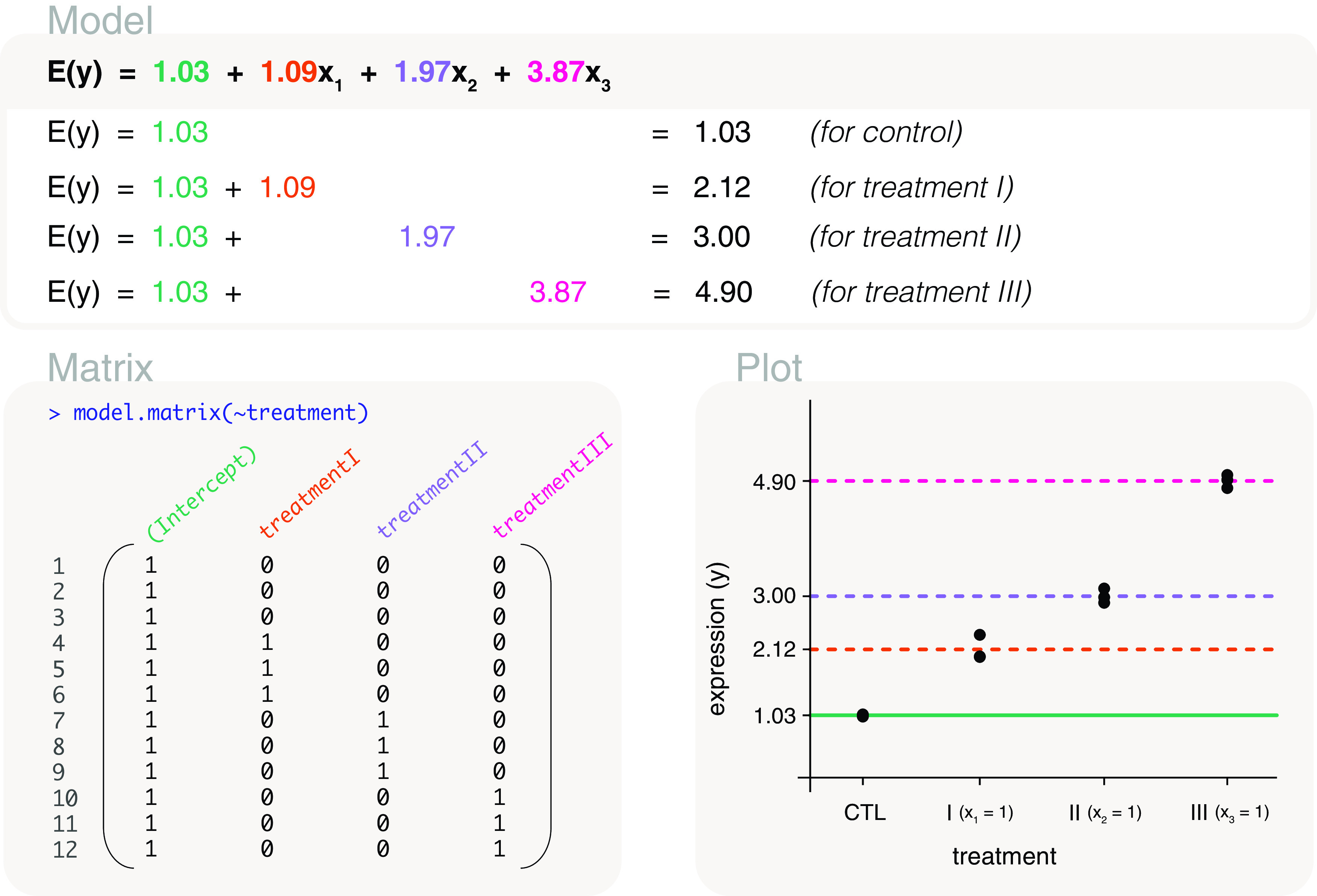
Expected gene expression is modelled by a treatment factor. The design matrix that is used includes an intercept term which represents the mean gene expression of the control group, or the reference level in the treatment factor. Other levels in the factor have mean gene expression represented relative to the control group. This means that the second to fourth parameters in the mean-reference model represent gene expression differences between treatment groups and the control group. The *x*’s in the model are indicator variables for treatment groups, with *x*_1_ = 1 for treatment I, *x*_2_ = 1 for treatment II, and *x*_3_ = 1 for treatment III.

We can now create a design matrix that represents a mean-reference model by model.matrix(~treatment) ( Figure 7). The columns of the design matrix represent the mean expression of the control group, and the difference in mean expression between treatment I and control, treatment II and control, and treatment III and control. Using the design matrix, the model parameters are estimated as 1.03, 1.09, 1.97 and 3.87. This means that the difference in expected gene expression between treatments and control are 1.09 for treatment I and control, 1.97 for treatment II and control, and 3.87 for treatment III and control. Treatment III has the greatest expected gene expression difference from the control. The fitted model for expected gene expression can then be written as *E*( *y*) = 1.03 + 1.09 *x*_1_ + 1.97 *x*_2_ + 3.87 *x*_3_, where the *x*’s are indicator variables for treatment I, treatment II and treatment III, respectively. In other words, *x*_1_ = 1 for treatment I, *x*_2_ = 1 for treatment II, and *x*_3_ = 1 for treatment III. The *x*’s are equal to 0 elsewhere.

### All pairwise comparisons

In order to make all possible pairwise comparisons between the treatments, we model gene expression using a means model. Due to its parameterisation, the means model is simple to work with when specifying the comparisons of interest in the contrast matrix.

**Figure 8.**
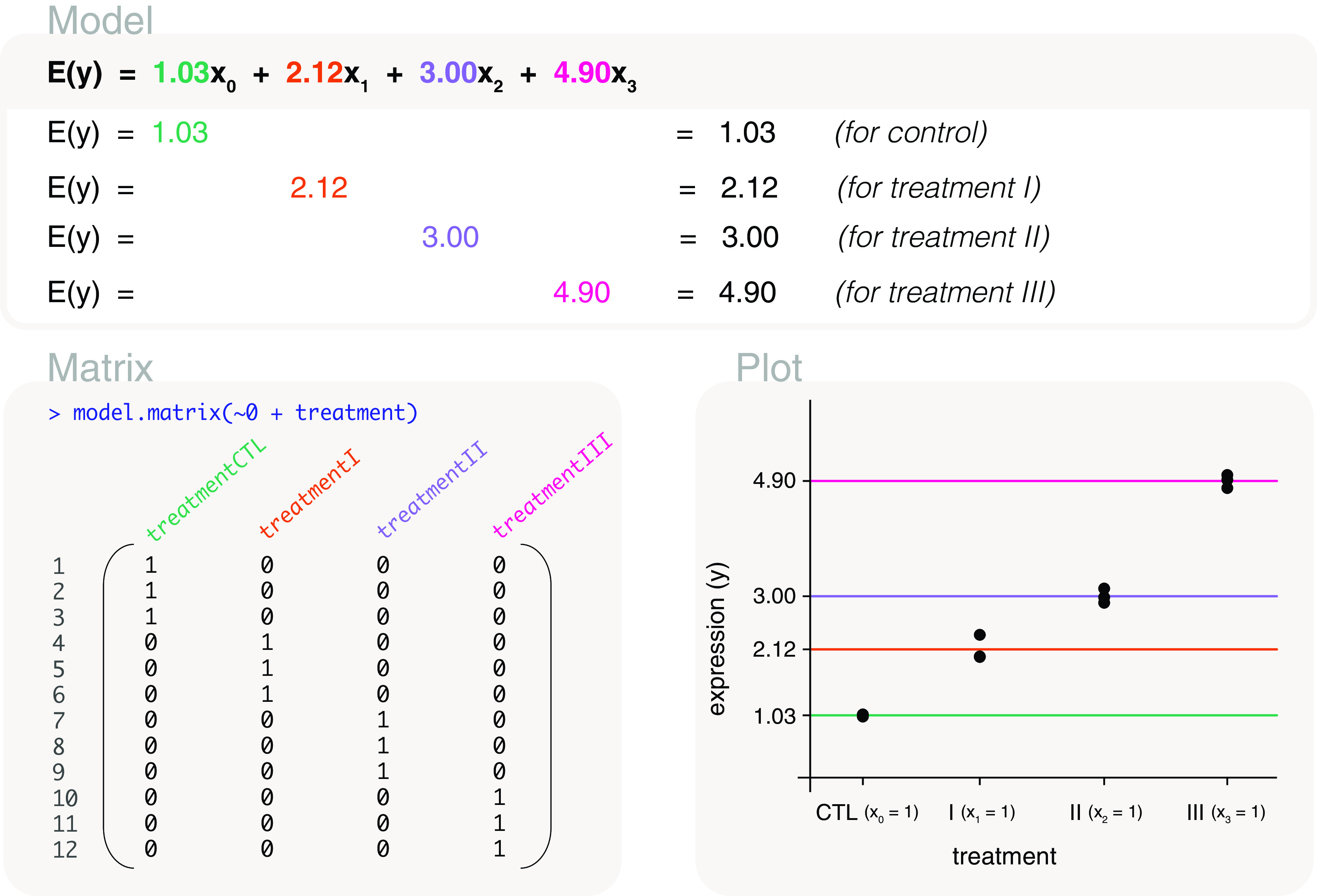
Expected gene expression is modelled by a treatment factor. The design matrix that is used excludes the intercept term so that the associated model is a means model. In other words, the mean gene expression of each level in ‘treatment’ is represented by a parameter in the model. The *x*’s in the model are indicator variables for control and treatment groups, with *x*_0_ = 1 for control, *x*_1_ = 1 for treatment I, *x*_2_ = 1 for treatment II, and *x*_3_ = 1 for treatment III.

The associated design matrix is coded as design <- model.matrix(~0+treatment), with columns or parameters representing the mean gene expression of each control and treatment group ( Figure 8). The mean expression values can then be estimated as 1.03, 2.12, 3 and 4.9; where the fitted model for expected gene expression is written as *E*( *y*) = 1.03 *x*_0_ + 2.12 *x*_1_ + 3 *x*_2_ + 4.9 *x*_3_. The *x*’s are indicator variables for control, treatment I, treatment II and treatment III, respectively. Specifically, *x*_0_ = 1 for control, *x*_1_ = 1 for treatment I, *x*_2_ = 1 for treatment II, and *x*_3_ = 1 for treatment III, and 0 elsewhere.

Taking these parameter estimates, we compute all pairwise differences between treatments using the makeContrasts function as follows:



contrasts <- makeContrasts(
  treatmentI-treatmentCTL, treatmentII-treatmentCTL, 
  treatmentIII-treatmentCTL, treatmentII-treatmentI, 
  treatmentIII-treatmentI, treatmentIII-treatmentII, 
  levels=colnames(design))
colnames(contrasts) <- abbreviate(colnames(contrasts))
contrasts

##               Contrasts
## Levels         tI-tC tII-tC tIII-tC tII-tI trIII-tI tIII-tII
##   treatmentCTL    -1     -1      -1      0        0        0
##   treatmentI       1      0       0     -1       -1        0
##   treatmentII      0      1       0      1        0       -1
##   treatmentIII     0      0       1      0        1        1



Note that there are six possible pairwise comparisons between the four treatments. Note also that default column names in the contrast matrix have been abbreviated here using the abbreviate function from the **base** package so that the contrast matrix can display neatly above (although this step is not usually necessary). The contrast matrix contains six columns, each representing one comparison: “tI-C” for treatment I versus control, “tII-C” for treatment II versus control, “tIII-C” for treatment III versus control, “tII-I” treatment II versus I, “trIII-I” for treatment III versus I, and “tIII-II” treatment III versus II. The 1s and -1s in each column of the contrast matrix mark the parameters from the design matrix from which comparisons are made. For example, the first contrast subtracts the mean expression of the control group (parameter 1) from the mean expression of treatment II (parameter 2), which is calculated as 1.09. In such a way, differences between treatments or between treatments and control are estimated. The difference in mean gene expression is estimated as 1.97 for treatment II versus control, 3.87 for treatment III versus control, 0.88 for treatment II versus I, 2.78 for treatment III versus I, and 1.9 for treatment III versus II.

### Control versus the rest

Rather than considering each treatment-control comparison separately, suppose that it is of interest to compare the control group to all of the treatment groups simultaneously. The idea of this is to find the genes that may define the control relative to the treatments. The same can also be carried out for individual treatment groups. For example, we could also consider the genes that define treatment I relative to the rest of the groups.

When comparing the control group to the rest of the groups, it is not advisable to merge treatments I, II and III into one big treatment group, and to simply fit a separate model for the combined treatment group and control. The combined treatment group does not account for group-specific variability, and the combined group would be biased towards larger treatment groups in an unbalanced study design. Instead, we demonstrate two methods to approach this. Both methods can use either of the fitted models from the previous sections (mean-reference or means model), where individual group means and variability are accounted for. The first method uses a contrast matrix to compare the control group to the treatment average, and the second looks at the overlap between treatment-control comparisons.

#### Control versus treatment average

Using the means model defined earlier, we calculate the average of the mean gene expression of treatment groups. We then subtract the mean gene expression of the control group from the average treatment value. To do this, a contrast matrix is coded as follows:



makeContrasts((treatmentI+treatmentII+treatmentIII)/3-treatmentCTL,
  levels=colnames(design))

##               Contrasts
## Levels         (treatmentI + treatmentII + treatmentIII)/3 - treatmentCTL
##   treatmentCTL                                                      -1.00
##   treatmentI                                                         0.33
##   treatmentII                                                        0.33
##   treatmentIII                                                       0.33



which calculates (2.12+3+4.9)/3 - 1.03 and is equal to 2.31. Notice how the parameter estimates for treatment groups are divided by 3, the number of treatment groups under consideration. This is important as it ensures the correct calculation of averages. What this method says is that the average gene expression of the treatment groups is greater than the control group by 2.31. In our case, the gene expression of each treatment group is also greater than the control. It is worth noting, however, that the average gene expression of the treatment groups can be greater than that of the control group when individual treatment groups are not necessarily all greater than the control.

#### Overlap of treatment-control results

For a more stringent approach that ensures that gene expression in each of the treatment groups are greater (or lower) than the control, we use a method of overlaps. Taking results from three treatment-control comparisons, we overlap or take the intersection of the genes that are significantly up-regulated (or down-regulated). Significance is usually defined by an adjusted *p*-value cut-off of 5%, but it can also be defined at varying thresholds or by using other summary statistics such as log-fold-changes. Notice that we take the direction of change into consideration so that genes are consistently up- or down-regulated in the control group. The direction of change can be determined by log-fold-change values, *t*-statistics or similar statistics. In the case where there are only a small number of significant genes in each of the treatment-control comparisons, the method described here can be overly stringent and result in no overlapping genes in the set. If this is the case, it would be reasonable to relax the threshold for defining significance.

### 2 versus 2 group comparisons

Let us suppose it is of interest to compare the gene expression of two groups against another two other groups. This may be of interest if there are prior expectations that two groups are more similar to each other than the other two. In this example, we compare control and treatment III against treatment I and II by applying the contrast coded as



makeContrasts((treatmentCTL+treatmentIII)/2-(treatmentI+treatmentII)/2, 
  levels=colnames(design))

##               Contrasts
## Levels         (treatmentCTL + treatmentIII)/2 - (treatmentI + treatmentII)/2
##   treatmentCTL                                                            0.5
##   treatmentI                                                             -0.5
##   treatmentII                                                            -0.5
##   treatmentIII                                                            0.5



to the means model. In defining the contrast, parameter estimates are divided out by the number of groups used to calculate the average. Using the parameter estimates, the difference in the 2 versus 2 group comparison is calculated as (1.03 + 4.9)/2 - (2.12 + 3)/2, which equals 0.41.

## Study of interactions and additivity of treatments

### Background

In this section, we reconsider the same experimental data as in the previous section, but we now suppose the treatment III is a combination of treatments I and II. Here we are interested in examining the effect of combining treatments I and II relative to their individual effects. We approach this using two methods. The first simply uses the parameter estimates that we have already calculated from the previous section, meaning that we use the single treatment factor to allocate sample information on the treatment and control types. The second approach uses two separate factors, which we will call treat1 and treat2, to allocate sample information on whether treatment I and/or treatment II were administered.

### Interaction using a single factor model

Using the first approach, we model the relationship between gene expression and the treatment factor with a mean-reference model. Taking the corresponding parameter estimates from the mean-reference model, such that we use the design matrix coded as model.matrix(~treatment), we find that the effect of treatment I relative to control is 1.09, such that the difference in means between treatment I and control is 1.09. The relative effect of treatment II is 1.97, and the relative effect of the combined treatment (previously referred to as treatment III) is 3.87. For simplicity, let us refer to these relative effects as *A*, *B* and *C*.

We consider the combined treatment to have an *additive* effect if the combined treatment effect is equal to the sum of the two individual effects, such that *C* − *A* − *B* = 0, which we simplify to *δ* = 0. On the other hand, we consider the combined treatment to have an *interaction* effect if the combined treatment effect is not equal to the sum of the two individual effects, such that *δ*≠0. An interaction effect is considered to be synergistic if the combined effect is greater than the sum of the individual effects ( *δ* > 0), and is considered repressive if the combined effect is less than the sum of the individual effects ( *δ* < 0). As you can see, it is of interest to determine the value of *δ*, which we call the interaction term. Using a design matrix with an intercept term, we define the interaction term, or *δ* = *C* − *A* − *B*, as a contrast in the makeContrast function, as follows:



design <- model.matrix(~treatment)
makeContrasts(treatmentIII-treatmentI-treatmentII,
  levels=colnames(design))

##               Contrasts
## Levels         treatmentIII - treatmentI - treatmentII
##   Intercept                                          0
##   treatmentI                                        -1
##   treatmentII                                       -1
##   treatmentIII                                       1



Taking the parameter estimates from the mean-reference model, this simply calculates *δ* as 3.87-1.09-1.97, which equals 0.82. Since the interaction term is a positive value, we conclude that combined treatment effect is interactive and synergistic.

Note that in running the makeContrasts function above, the function automatically converted the “(Intercept)” column in the design matrix to “Intercept” since the brackets are syntactically invalid. To avoid distracting from the results, we suppressed the display of its warning message referring to this in our output above.

### Interactions using a two-factor model

Another way to approach the same problem is by reassign the explanatory variable into two factors representing the presence and absence of the treatments. The factors treat1 and treat2 are defined as follows:



##    expression   mouse treat1 treat2
## 1        1.01  MOUSE1     NO     NO
## 2        1.04  MOUSE2     NO     NO
## 3        1.04  MOUSE3     NO     NO
## 4        1.99  MOUSE4    YES     NO
## 5        2.36  MOUSE5    YES     NO
## 6        2.00  MOUSE6    YES     NO
## 7        2.89  MOUSE7     NO    YES
## 8        3.12  MOUSE8     NO    YES
## 9        2.98  MOUSE9     NO    YES
## 10       5.00 MOUSE10    YES    YES
## 11       4.92 MOUSE11    YES    YES
## 12       4.78 MOUSE12    YES    YES



**Figure 9.**
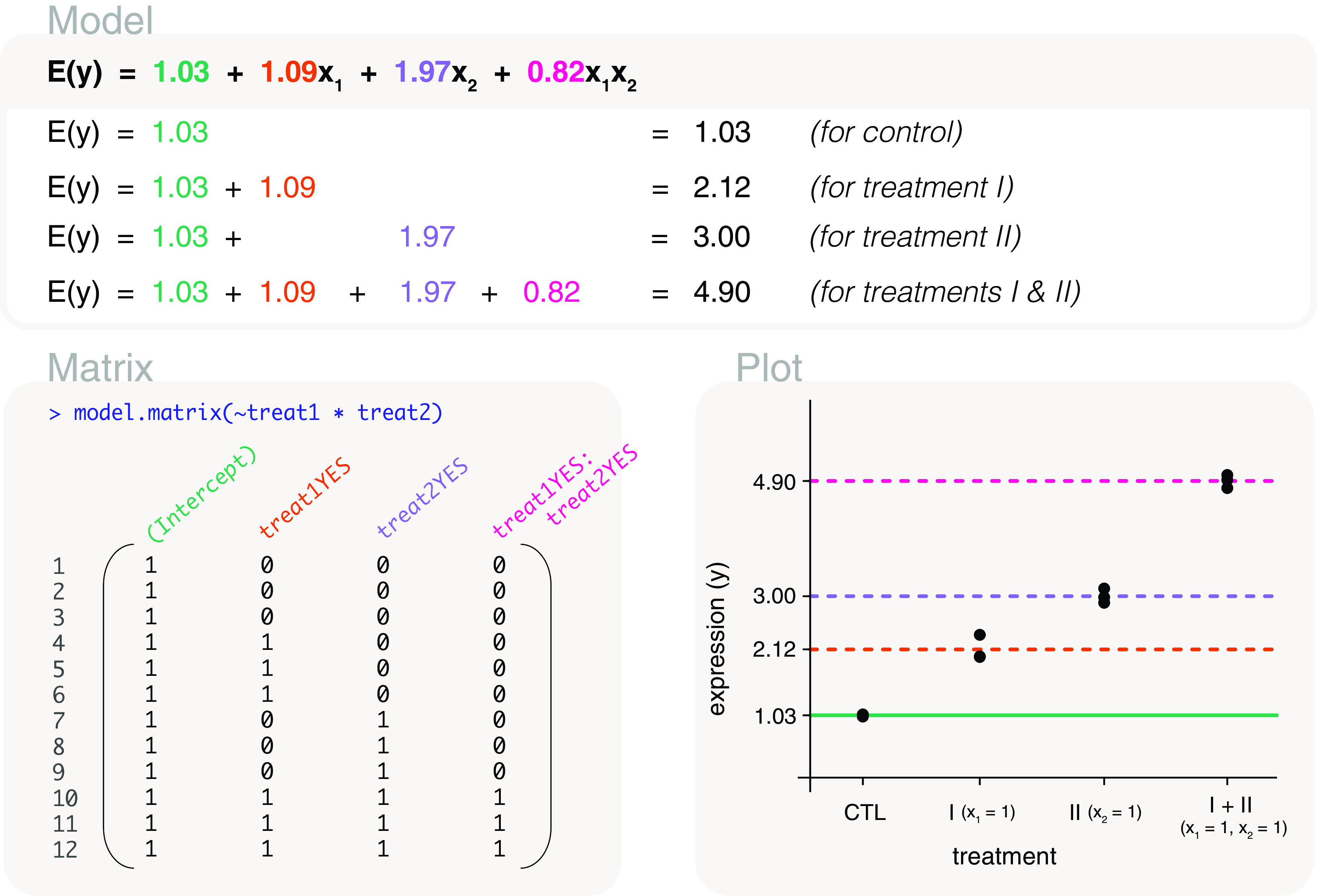
Expected gene expression is modelled by a two factors representing treatment I and treatment II. The design matrix that is used includes an interaction term in the last column, and we refer to the associated model as an interaction model. The interaction term can be used to indicates whether the combined administration of treatments I and II have an additive effect (interaction term equal to zero), have a synergistic effect (interaction term has a positive value), or have a repressive effect (interaction term has a negative value). In this example, the interactive effect is estimated as 0.82. The *x*’s in the model are indicator variables for treatment I and treatment II, where *x*_1_ *x*_2_ is only equal to 1 if both treatments are present.

Here treat1 indicates the presence or absence of treatment I and treat2 indicates the presence or absence of treatment II. The two factors allow us to create a model that directly includes an interaction term. The associated design matrix is coded as model.matrix(~treat1*treat2), where an asterisk is placed between the two factors ( Figure 9). The design matrix is parameterised for the mean gene expression of the control group (first column), the difference in mean expression between treatment I and control (second column), treatment II and control (third column), and the interaction term (last column). Using this design matrix, the parameters can be estimated as 1.03, 1.09, 1.97 and 0.82. In other words, the interaction term is estimated as 0.82, and has the same value as calculated previously.

Moreover, whether we use a single treatment factor or the two factors here, the two models are equivalent, differing only in parameterisation. The interaction model fitted here can be written as *E*( *y*) = 1.03 + 1.09 *x*_1_ + 1.97 *x*_2_ + 0.82 *x*_1_ *x*_2_, where the *x*_1_ and *x*_2_ are indicator variables for treatment I and treatment II respectively. Specifically, the fourth term in the model, the interaction term, is only included in the presence of both treatments, such that *x*_1_ *x*_2_ = 1 but is 0 elsewhere.

### Additivity using a two-factor model

Whilst the interaction model is useful in identifying the effect of the combined treatment via the interaction term, such a model may not always be of interest. One may simply want to quantify the individual effects of treatment I and treatment II, and prefer the assumption that a combined treatment results in the additivity of the two effects. This means that we use all of the samples associated with treatment I (treatment I only and in combination with treatment II) to estimate the effect of treatment I. The same goes for treatment II.

**Figure 10.**
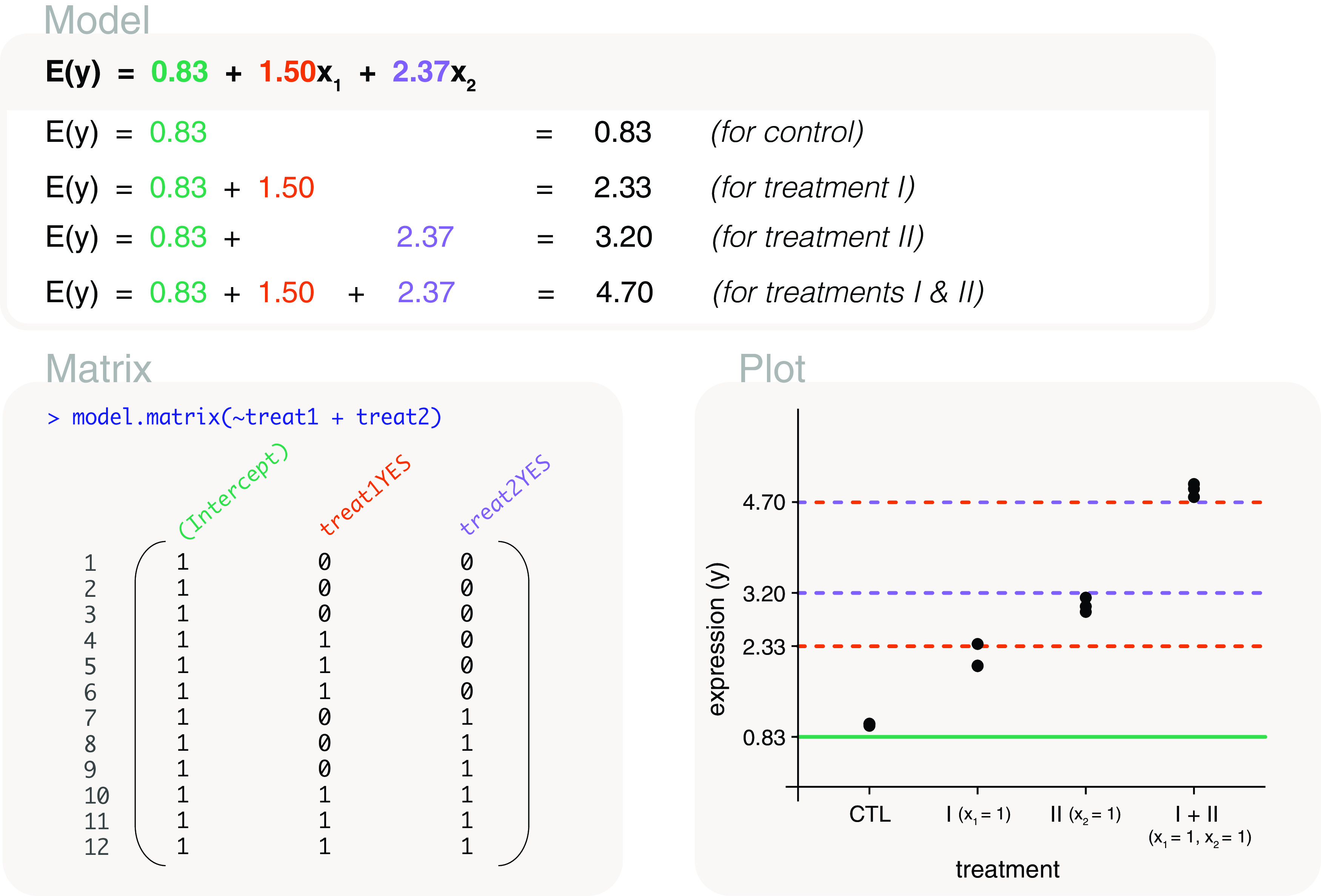
Expected gene expression is modelled by a two factors representing treatment I and treatment II. The design matrix that is used excludes the interaction term, and we refer to the associated model as an additive model. This means that a combined treatment is assumed to have the additive effects of individual treatment I and treatment II effects. The *x*’s in the model are indicator variables for treatment I and treatment II.

Using the two factors treat1 and treat2, we create an additive model that excludes the interaction term. The associated design matrix is coded as model.matrix(~treat1+treat2), where a plus sign is placed between the two factors ( Figure 10). The design matrix contains 3 columns that are identical to the first three columns of the design matrix from the interaction model ( Figure 9). The interpretation of those parameters remain the same. The first parameter represents the mean expression of the control group, the second represents the difference in mean expression between treatment I and control, and the third parameter represents the difference in mean expression between treatment II and control. The parameter estimates for this model can be calculated as 0.83, 1.5 and 2.37. The mean expression of the combined treatment can be calculated by combining all parameter estimates (gene expression from the control group, and the relative change when treatments I and II are added), such that it is equal to 0.83+1.5+2.37=4.7. We can write the fitted additive model as *E*( *y*) = 0.83 + 1.5 *x*_1_ + 2.37 *x*_2_, where the *x*_1_ and *x*_2_ are indicator variables for treatment I and treatment II. Relative to the interaction model, the fit of the additive model results in expected gene expression values that are further from the observed values. This is not unexpected since fewer parameters are used to model the relationship between gene expression and groups, and we know from the interaction model that the interaction term is non-zero. Even so, the additive model may be preferred for its simple interpretation and thus may be more applicable to some studies.

## Studies with multiple factors

### Background

In this section, we examine several study designs that contain two or more factors as explanatory variables. We begin with an example where we convert two factors of interest into one, and then consider cases where there are factors that are not of interest. In the second half of this section, more complex study designs are introduced, such as scenarios where there are nested factors and repeated measurements. We finish off the section by fitting a mixed effects model using functions from the **limma** package, where we treat a factor that is not of interest to the study as a random effect.

### Conversion to a single factor

Experimental studies often include multiple factors of interest. This could involve different treatments, cell types, tissue types, sex, and so on. Let us consider an experiment on lung and brain samples that are enriched for B-cells and T-cells. The data is as follows:



##    expression      id tissue cells
## 1        1.01  MOUSE1   LUNG     B
## 2        1.04  MOUSE2   LUNG     B
## 3        1.04  MOUSE3   LUNG     B
## 4        1.99  MOUSE4  BRAIN     B
## 5        2.36  MOUSE5  BRAIN     B
## 6        2.00  MOUSE6  BRAIN     B
## 7        2.89  MOUSE7   LUNG     T
## 8        3.12  MOUSE8   LUNG     T
## 9        2.98  MOUSE9   LUNG     T
## 10       5.00 MOUSE10  BRAIN     T
## 11       4.92 MOUSE11  BRAIN     T
## 12       4.78 MOUSE12  BRAIN     T



For this experiment, there are several comparisons of interest: 1) overall differences between cell types, 2) overall differences between tissues, 3) differences between cell types within each tissue type, and 4) differences between tissues within each cell type. The simplest method is to merge tissue and cells factors into a single group factor, as follows:



##    expression      id tissue cells   group
## 1        1.01  MOUSE1   LUNG     B  LUNG_B
## 2        1.04  MOUSE2   LUNG     B  LUNG_B
## 3        1.04  MOUSE3   LUNG     B  LUNG_B
## 4        1.99  MOUSE4  BRAIN     B BRAIN_B
## 5        2.36  MOUSE5  BRAIN     B BRAIN_B
## 6        2.00  MOUSE6  BRAIN     B BRAIN_B
## 7        2.89  MOUSE7   LUNG     T  LUNG_T
## 8        3.12  MOUSE8   LUNG     T  LUNG_T
## 9        2.98  MOUSE9   LUNG     T  LUNG_T
## 10       5.00 MOUSE10  BRAIN     T BRAIN_T
## 11       4.92 MOUSE11  BRAIN     T BRAIN_T
## 12       4.78 MOUSE12  BRAIN     T BRAIN_T



**Figure 11.**
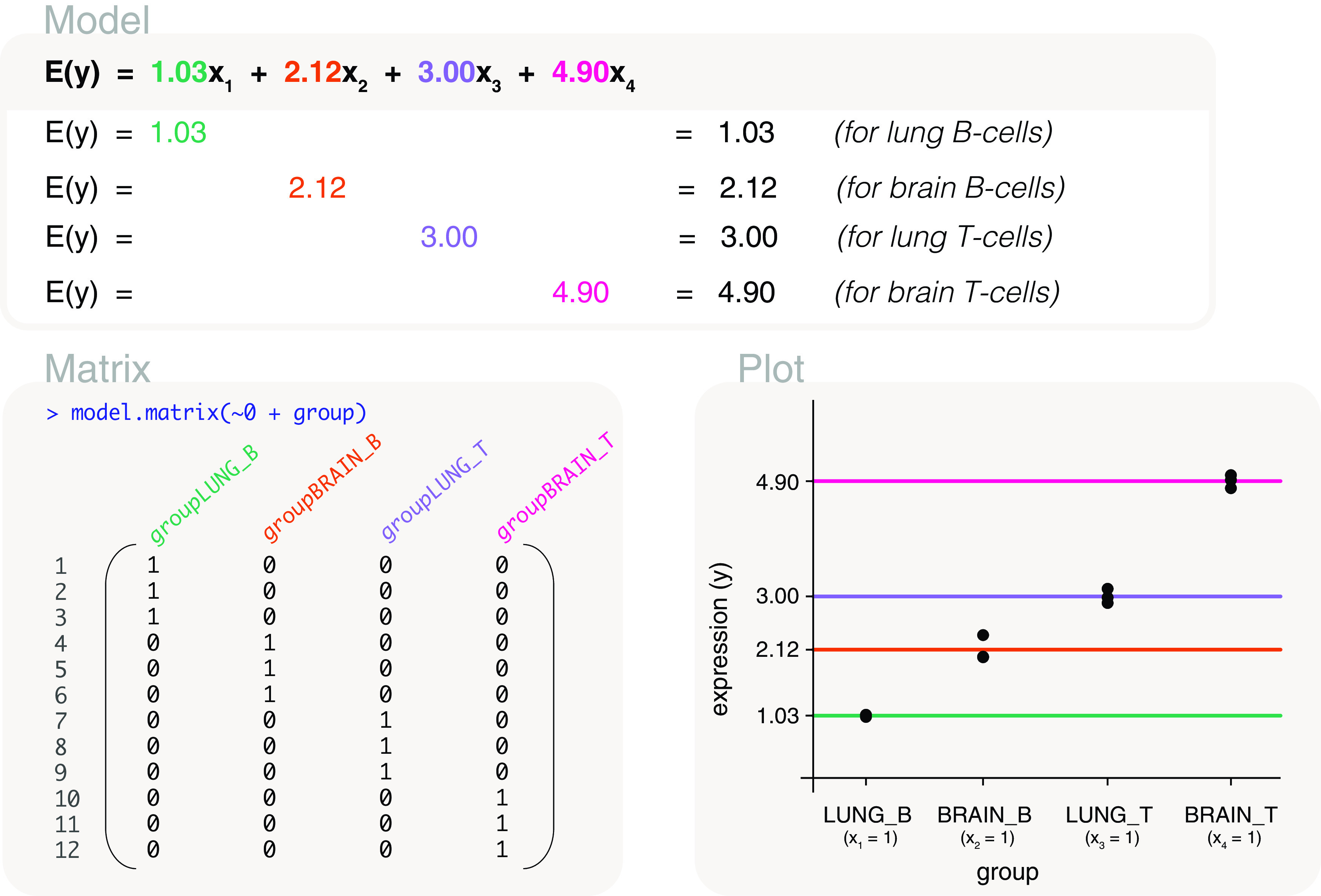
Expected gene expression is modelled by a single group factor using a design matrix that excludes the intercept term. The group factor is converted from two factors representing tissue samples and cell types.

This allows us to fit a means model to the data, using a design matrix coded as design <- model.matrix(~0+group) ( Figure 11), and to define contrasts for comparisons of interest using the makeContrasts function. The contrasts are coded as



contrasts <- makeContrasts(
  BVsT=(groupLUNG_B+groupBRAIN_B)/2-(groupLUNG_T+groupBRAIN_T)/2,
  LungVsBrain=(groupLUNG_B+groupLUNG_T)/2-(groupBRAIN_B+groupBRAIN_T)/2,
  BVsT_Lung=groupLUNG_B-groupLUNG_T,
  BVsT_Brain=groupBRAIN_B-groupBRAIN_T,
  LungVsBrain_B=groupLUNG_B-groupBRAIN_B, 
  LungVsBrain_T=groupLUNG_T-groupBRAIN_T, 
  levels=colnames(design))
rownames(contrasts) <- gsub("group", "", rownames(contrasts))
contrasts

##          Contrasts
## Levels    BVsT LungVsBrain BVsT_Lung BVsT_Brain LungVsBrain_B LungVsBrain_T
##   LUNG_B   0.5         0.5         1          0             1             0
##   BRAIN_B  0.5        -0.5         0          1            -1             0
##   LUNG_T  -0.5         0.5        -1          0             0             1
##   BRAIN_T -0.5        -0.5         0         -1             0            -1



with columns of the matrix representing 1) overall differences between cells, B-cells versus T-cells; 2) overall differences between tissues, lung versus brain; 3) differences between cells within lung, and 4) differences between cells within brain; 5) differences between tissues within B-cells, and 6) differences between tissues within T-cells. Notice that we specified our own contrast names in the code above. The row names were also shortened so that the contrast matrix could display neatly.

Using the design matrix, the parameters are estimated as 1.03 for the mean gene expression of B-cells in the lung, 2.12 for B-cells in the brain, 3 for T-cells in the lung, and 4.9 for T-cells in the brain. By applying the contrast matrix to the estimated parameters, we calculate that overall gene expression difference between B-cells versus T-cells is -2.37, and -1.5 for lung versus brain. B-cells and T-cells differ by -1.97 in the lung, and -2.78 in the brain. Lung samples and brain samples differ by -1.09 in B-cells, and by -1.9 in T-cells.

### Accounting for factors that are not of interest

Some factors within an experiment may not be of biological interest. Often they are technical factors such as handling technician, experimental time if samples were processed in separate batches, or the sequencing lane on which the samples were processed on. There are also biological factors that may not be of direct interest; such as ethnicity of patients in a human drug trial or the sex of individuals from which samples were taken. Let us consider an experiment with mice belonging to groups A, B, C, or D, each in triplicate. It is of interest to compare gene expression between the groups. In the process of the experiment, two sequencing lanes (L1 and L2) were used for sequencing and samples were processed by different technicians (I and II). To ensure that differences detected between groups are not influenced by these factors, we can account for any differences between the sequencing lanes and handling technician in our modelling process. The data is as follows:



##    expression      id group lane technician
## 1        1.01  MOUSE1     A   L2          I
## 2        1.04  MOUSE2     A   L2          I
## 3        1.04  MOUSE3     A   L1         II
## 4        1.99  MOUSE4     B   L1          I
## 5        2.36  MOUSE5     B   L2         II
## 6        2.00  MOUSE6     B   L1          I
## 7        2.89  MOUSE7     C   L1         II
## 8        3.12  MOUSE8     C   L2         II
## 9        2.98  MOUSE9     C   L1          I
## 10       5.00 MOUSE10     D   L1         II
## 11       4.92 MOUSE11     D   L2         II
## 12       4.78 MOUSE12     D   L2          I



**Figure 12.**
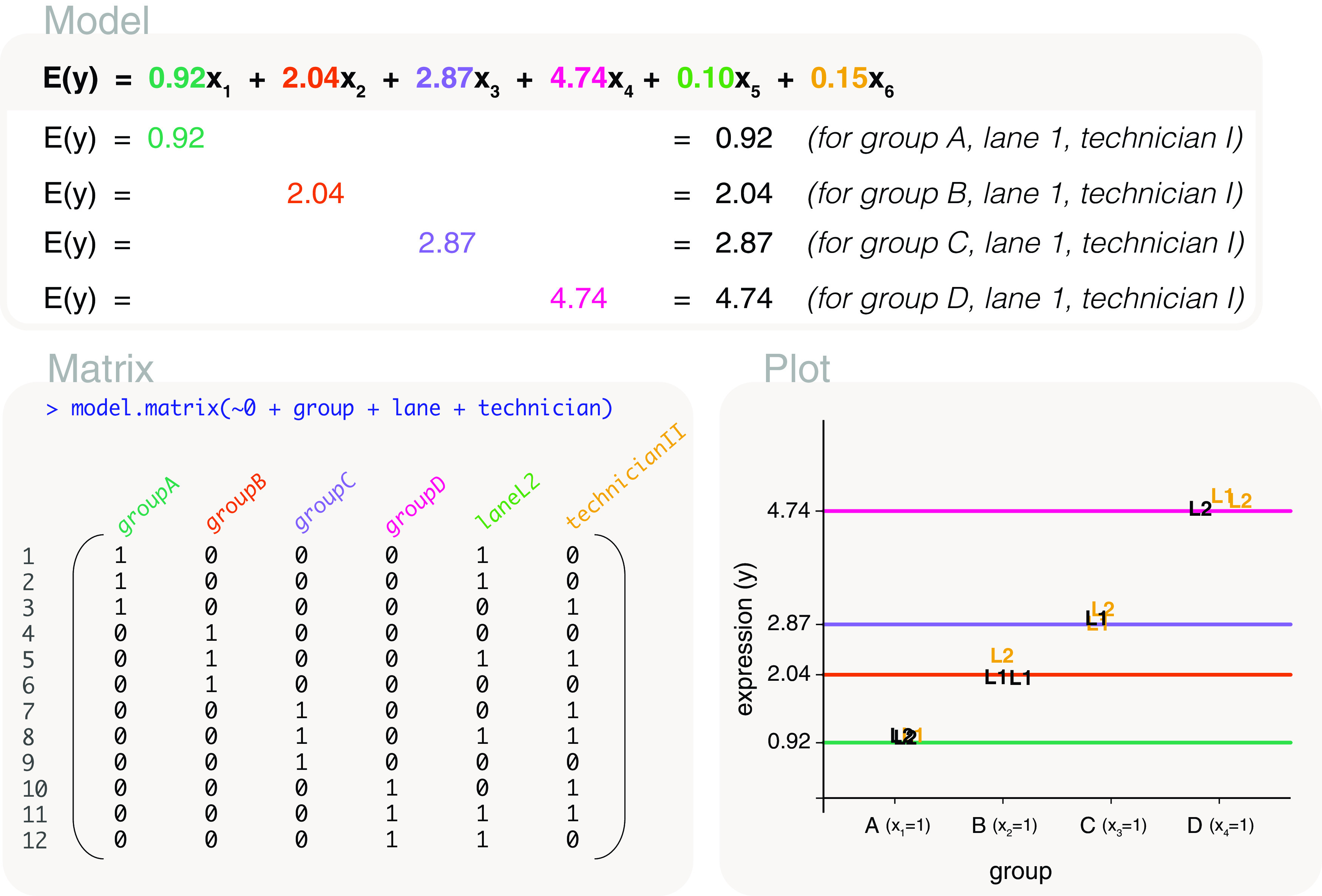
Expected gene expression is modelled by a group factor and two additional factors that are not of interest (lane and technician). The design matrix excludes the intercept term for the first factor added to the function. Only lines reflecting the first 4 parameters are drawn in the plot, representing the mean gene expression of groups A, B, C and D in lane L1 and with handling technician I. Samples are labelled by their sequencing lane (L1 or L2), and coloured black if they are processed by technician I, yellow if they are processed by technician II.

A means model can be fitted to the data, with a design matrix coded as design <- model.matrix(~0+group+lane+technician) to model gene expression in groups, while accounting for effects resulting from differences in lane and technician. The first 4 columns of the design matrix are associated with parameters for the mean expression of group A, B, C and D ( Figure 12). Specifically, the group means are parameterised for when the samples are in lane L1 and processed by technician I.

The fifth column in the design matrix is parameterised for difference between lane L2 and lane L1 (for group A samples processed by technician I), and the sixth column is parameterised for the difference between technician II and I (for group A samples in lane L1). Although an intercept-free design matrix has been coded using the 0+ notation, the intercept is only excluded from the first factor that is listed within the model.matrix function. In other words, the second and third factors added to the model.matrix function are parameterised as though there is an intercept term. This is why we place the factor of interest first as it simplifies the subsequent code for the comparisons of interest, even though a different order of factors added give equivalent models with variations in parameterisation.

By estimating model parameters, the fitted model can be written as *E*( *y*) = 0.92 *x*_1_ + 2.04 *x*_2_ + 2.87 *x*_3_ + 4.74 *x*_4_ + 0.1 *x*_5_ + 0.15 *x*_6_, where *x*_1_ to *x*_4_ are indicator variables for groups A to D, respectively. Additionally, *x*_5_ is an indicator variable for lane L2, and *x*_6_ is an indicator variable for technician II. In other words, a group A sample processed in lane L1 and by technician I has expected gene expression *E*( *y*)=0.92. Whereas, the expected gene expression is *E*( *y*)=0.92+0.1=1.02 if it were processed in lane L2, *E*( *y*)=0.92+0.15=1.06 for technician II, and *E*( *y*)=0.92+0.1+0.15=1.16 for a group A sample processed in lane L2 and by technician II.

For comparisons between groups, we form contrasts using only the first 4 parameter estimates, and keep lane and technician consistent. For example, a contrast comparing group A to group B can be coded as makeContrasts(groupA-groupB, levels=colnames(design)). All other pairwise comparisons can also be included into the contrast matrix.

When modelling multiple factors of interest, the factors may be converted into a single factor for modelling, as shown in the previous section. We also note that it may not be sensible to add all known factors associated with the experiment. This could well exceed the number of degrees of freedom available for modelling (too many parameters when compared to the number of data points). A reasonable way to check the factors that should be accounted for include the use of unsupervised clustering plots, such as principal components analysis (PCA) or multi-dimensional scaling (MDS). Factors associated with separation between sample clusters should be included in the model. An alternative method is to fit a model to the biological groups of interest with one addition factor to observe whether the factor has substantial influence on gene expression (such that many genes are detected as differentially expressed for that factor). Repeat this for subsequent factors to determine the factors that should be included into the final model.

### Nested factors and matrices without full rank

Now consider a study design that includes two of the factors, group and batch, representing biological groups of interest and experimental batches. The samples in group A and group B are processed in batch B1, whilst samples in group C and group D are processed in batch B2. We say that the groups are *nested* within batches. The data is as follows:



##    expression      id group batch
## 1        1.01  MOUSE1     A    B1
## 2        1.04  MOUSE2     A    B1
## 3        1.04  MOUSE3     A    B1
## 4        1.99  MOUSE4     B    B1
## 5        2.36  MOUSE5     B    B1
## 6        2.00  MOUSE6     B    B1
## 7        2.89  MOUSE7     C    B2
## 8        3.12  MOUSE8     C    B2
## 9        2.98  MOUSE9     C    B2
## 10       5.00 MOUSE10     D    B2
## 11       4.92 MOUSE11     D    B2
## 12       4.78 MOUSE12     D    B2



It is of interest to compare the gene expression between groups. Naturally, one may include both factors into a design matrix coded as design <- model.matrix(~0+group+batch) or design <- model.matrix(~0+batch+group). This, however, produces a design matrix that is not of full rank, meaning that there are more columns in the design matrix (5 columns in this case) than what is needed (4 columns). The resultant design matrix has some columns that are linearly dependent, which is due to batch information being redundant once all group means are defined. This is because batch B1 is uniquely defined by group A and B, and batch B2 is uniquely defined by group C and D. Similarly, two of the groups are redundant if batch means are defined first. One would usually notice that their design matrix is not of full rank when the parameter estimation process returns NA or non-estimable results for some parameters.

To check for redundancy of model parameters, one can compare between the number of columns in the design matrix with ncol(design) to the rank of the matrix with qr(design)$rank. This would show that there are 5 columns in the design matrix but only a rank of 4, meaning that one of the parameters defined in the design matrix is linearly dependent. This should prompt us to consider how to set up the model properly, figuring out which factors are dependent on others, and ultimately redefining the design matrix. For example, the design matrix can be set to model.matrix(~0+group) instead, although we should keep in mind that some pairwise group comparisons would be confounded by batch effects, such as when comparing group A to group C.

### Time series experiment with repeated mouse measurements nested within treatments

In a study of treatment effects, gene expression measurements were taken from mice at multiple time points. Three of the mice were administered treatment X, and another three were administered treatment Y. Measurements were taken for the mice at two timepoints, T1 (baseline) and T2. The data is as follows:



##    expression     id treatment timepoint
## 1        1.01 MOUSE1         X        T1
## 2        1.04 MOUSE2         X        T1
## 3        1.04 MOUSE3         X        T1
## 4        1.99 MOUSE1         X        T2
## 5        2.36 MOUSE2         X        T2
## 6        2.00 MOUSE3         X        T2
## 7        2.89 MOUSE4         Y        T1
## 8        3.12 MOUSE5         Y        T1
## 9        2.98 MOUSE6         Y        T1
## 10       5.00 MOUSE4         Y        T2
## 11       4.92 MOUSE5         Y        T2
## 12       4.78 MOUSE6         Y        T2



It is of interest to compare timepoint T1 with T2 within treatment X, while accounting for how the samples are paired. What this means is that it is important to account for the relative change from timepoint T1 to T2 of each mice, when estimating the overall change between the timepoints. Similarly, a comparison between timepoint T1 and T2 within treatment Y is of interest. Additionally, we want to examine the overall differences between treatment X and Y. Since the mice are nested within treatment types, we create a custom design matrix to avoid a matrix that has linearly dependent columns or that is not of full rank. The custom design matrix is created using model.matrix and cbind functions in the following way:



design <- model.matrix(~0+id)
design <- cbind(design, X= treatment=="X" & timepoint=="T2")
design <- cbind(design, Y= treatment=="Y" & timepoint=="T2")



**Figure 13.**
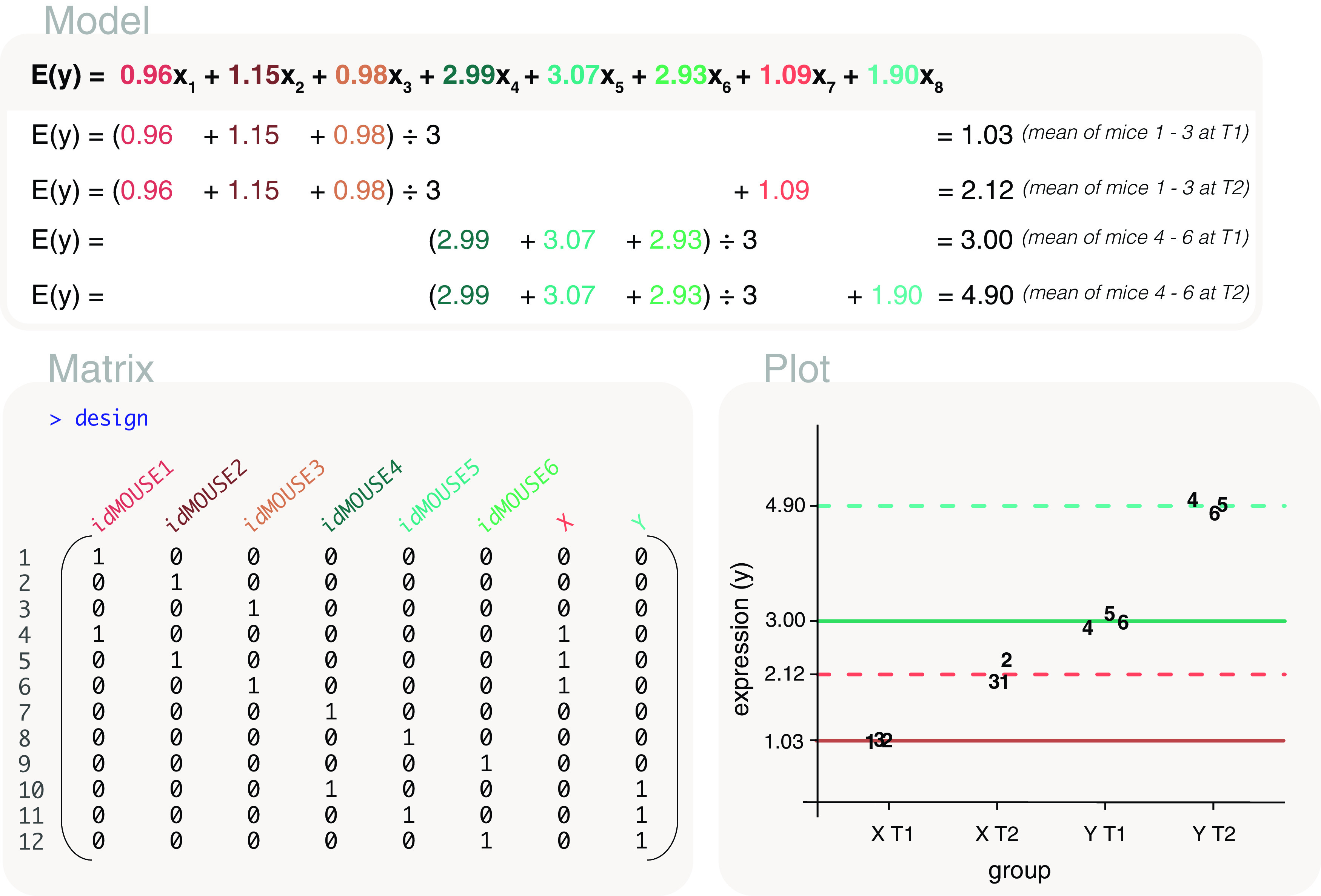
We model the expected gene expression of mice that have been given either treatment X or treatment Y, with samples taken at timepoints T1 and T2. Repeated measurements are taken from mice, as indicated by the numbers in the plot, such that label ”1” represents MOUSE1. A custom design matrix is created to model mouse IDs, treatment and timepoint (complete R code shown in the main article). Fitted lines are drawn in pink for treatment X, and in aqua for treatment Y. Solid lines represent expected gene expression at timepoint T1, and dashed lines for timepoint T2.

In the first step, model.matrix(~0+id), we account for each individual mouse and the pairing of samples. Since mice are nested within treatments, the treatment effects are encompassed within the mouse effects. In the second step, cbind(design, X= treatment=="X" & timepoint=="T2"), an extra “X” column is added to the design matrix to represent treatment X at timepoint T2. Similarly, the third step, cbind(design, Y= treatment=="Y" & timepoint=="T2"), appends column “Y” to the design matrix to represent treatment Y at timepoint T2. The two additional columns differentiate between samples at timepoint T1 (first 6 columns) from those at timepoint T2 (last 2 columns), such that the first 6 columns in the design matrix now represent the effect of each mouse at timepoint T1, and the last 2 columns represent the overall difference between timepoint T2 and T1 for treatments X and Y respectively ( Figure 13).

The first 6 parameters, which represent the expected expression of each mouse at timepoint T1, can be estimated as 0.96, 1.15, 0.98, 2.99, 3.07 and 2.93. Using the first three estimates (0.96, and 1.15, 0.98), we can calculate the expected expression of treatment X at timepoint T1 by taking the mean value, which equals 1.03 (and is marked by the thick, dark red line in the plot of Figure 13). Similarly, the next three estimates (2.99, 3.07 and 2.93) can be used to calculate the expected expression of treatment Y at timepoint T1, which equals 3 (and is marked by the thick, dark green line in the plot of Figure 13). The seventh parameter is estimated at 1.09, and the eighth is 1.9, such that gene expression is greater by 1.09 and 1.9 at timepoint T2 relative to timepoint T1 for treatments X and Y respectively. The overall difference between treatments X and Y can be coded as makeContrasts(X-Y, levels=colnames(design)), which calculates the difference between the seventh and eighth parameters, and has a value of -0.82.

### Treating factors that are not of direct interest as random effects

In the previous section, repeated measurements taken from mice receiving treatment X or Y were accounted for within the design matrix. By including the mouse IDs into the design matrix, we say that mouse IDs were treated as fixed effects in the modelling process. An alternative method treats the mouse IDs instead as random effects, and does not include the IDs into the design matrix. We refer to this type of model as a *mixed effects model*, such that treatment and timepoint are included into the design matrix as fixed effects in the model, whilst mouse IDs are included as random effects. One important advantage to the **limma** package is that it has the ability to fit a mixed effects model, unlike **edgeR** or **DESeq2**, which can only fit fixed effects.

Why do we fit mouse IDs as a random effect rather than a fixed effect? The specific differences between mice are not of direct interest to the study, so removing them from the design matrix reduces the number of model parameters, conserves the number of degrees of freedom in modelling, and likely increases statistical power for testing. The effects, however, cannot be omitted completely because they are integral to the study design; individual mouse effects should still be accounted for when calculating relative difference between timepoints T1 and T2.

To fit a mixed effects model, let us first define our fixed effects in a design matrix. To simplify the two factors of interest, treatment and timepoint, we merge them into a single factor called group. The data which includes the new group factor are as follows:



##    expression     id treatment timepoint group
## 1        1.01 MOUSE1         X        T1  X_T1
## 2        1.04 MOUSE2         X        T1  X_T1
## 3        1.04 MOUSE3         X        T1  X_T1
## 4        1.99 MOUSE1         X        T2  X_T2
## 5        2.36 MOUSE2         X        T2  X_T2
## 6        2.00 MOUSE3         X        T2  X_T2
## 7        2.89 MOUSE4         Y        T1  Y_T1
## 8        3.12 MOUSE5         Y        T1  Y_T1
## 9        2.98 MOUSE6         Y        T1  Y_T1
## 10       5.00 MOUSE4         Y        T2  Y_T2
## 11       4.92 MOUSE5         Y        T2  Y_T2
## 12       4.78 MOUSE6         Y        T2  Y_T2



**Figure 14.**
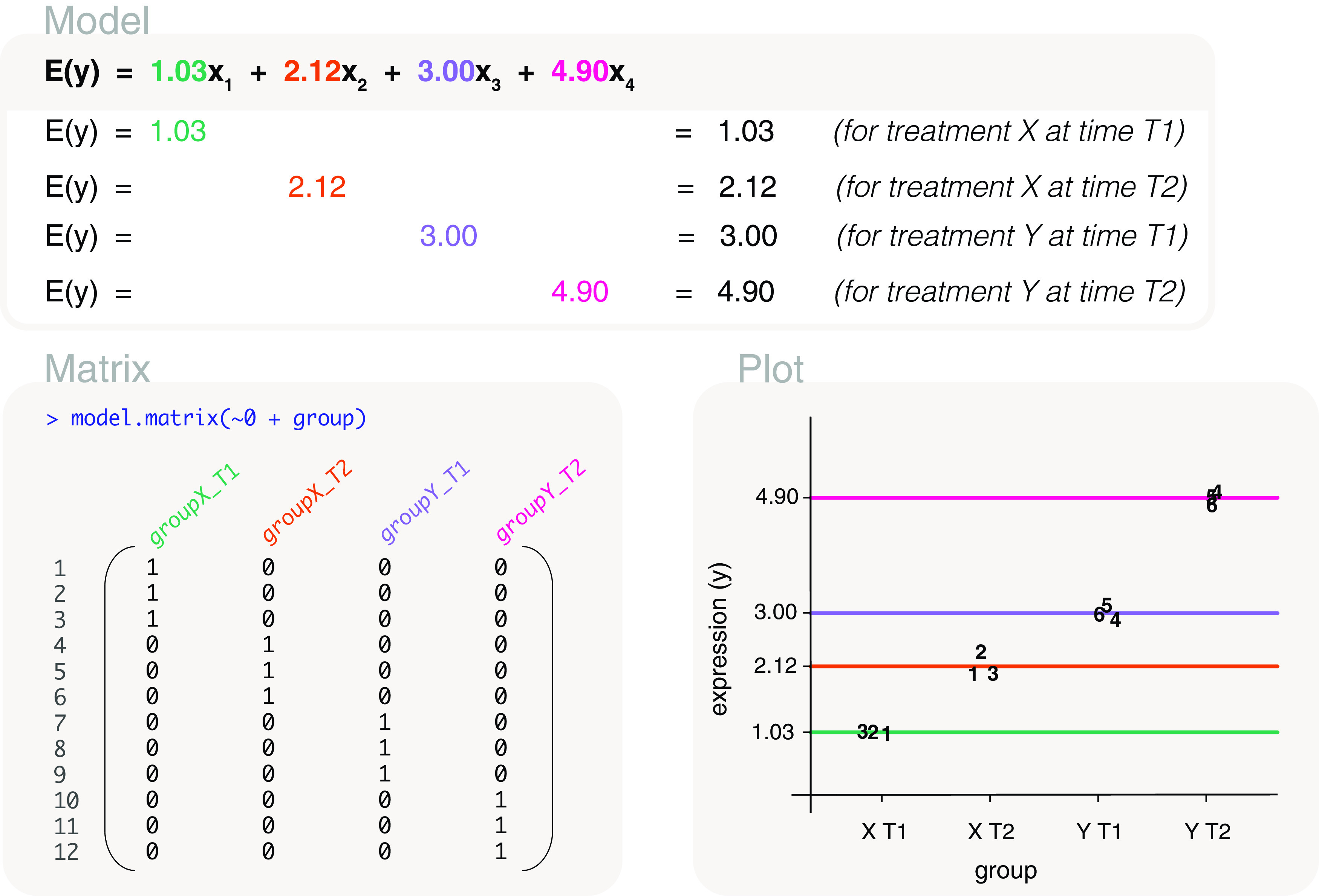
Expected gene expression is modelled by a mixed effects model, with treatment and timepoint (converted into a single group factor) as fixed effects and mouse IDs as the random effects. A means model is fitted to the data using a design matrix that excludes the intercept term. In the plot, data points are labelled by mouse ID.

A means model is fitted to the groups by coding the design matrix as design <- model.matrix(~0+group), which gives a 4 column matrix representing the mean gene expression in each group ( Figure 14). Mouse IDs are set as random effects by assigning id as the blocking variable in the lmFit function in **limma**. Before doing this, we first estimate the correlation between repeated mice measurements using the duplicateCorrelation function in **limma** as follows:



cor <- duplicateCorrelation(expression, design, block=id)
cor$consensus.correlation

## [1] -0.05



The correlation between measurements taken from the same mouse is estimated as -0.05, which is considered to be quite a small correlation value. This is expected in our example since we did not program specific mouse effects into the dataset. In the case of a negative estimated correlation, the blocking variable should be removed and we can resume the usual modelling approach of accounting for the group fixed effect only in the design matrix. In fact, the blocking variable can be removed if the estimated correlation is very, very small (say less than 0.01) as its contribution to the overall model fit would also be very minor, however, keeping it in the model would not adversely affect the modelling process either. For real use cases, correlation estimates of 0.7 to 0.9 are considered high but not uncommon. Despite our recommendation above, let us continue with the fitting of our mixed effects model for the sake of demonstrating how it can be carried out.

Using the lmFit function, we fit our random effects by setting the correlation argument to the estimated correlation and block argument to mouse id. The fixed effects modelled within the design matrix are given to the design argument, along with the expression data as follows:



fit <- lmFit(object=expression, design=design, 
  block=id, correlation=cor$consensus.correlation)



The mixed effects model estimates the mean gene expression of mouse receiving treatment X at timepoint T1 to be 1.03, treatment X at timepoint T2 to be 2.12, treatment Y at timepoint T1 to be 3, and treatment Y at timepoint T1 to be 4.9. To obtain estimates for the comparisons of interest, we use a contrast matrix coded as:



contrasts <- makeContrasts(
  X_T2vsT1=groupX_T2-groupX_T1, 
  Y_T2vsT1=groupY_T2-groupY_T1, 
  XvsY=(groupX_T2-groupX_T1)-(groupY_T2-groupY_T1), 
  levels=colnames(design)) 
contrasts

##            Contrasts
## Levels      X_T2vsT1 Y_T2vsT1 XvsY
##   groupX_T1       -1        0   -1
##   groupX_T2        1        0    1
##   groupY_T1        0       -1    1
##   groupY_T2        0        1   -1



The first contrast calculates the difference between timepoint T2 and T1 in treatment X, the second contrast calculates the difference between timepoint T2 and T1 in treatment Y, and the last contrast the overall difference between treatment X and Y. The overall difference between treatment X and Y is calculated after adjusting the mean gene expression at timepoint T2 by that of the baseline (timepoint T1). The contrast matrix is incorporated into the **limma** pipeline using the contrasts.fit function as follows:



fit <- contrasts.fit(fit, contrasts)



Using the contrast matrix, the difference between timepoint T2 and T1 in treatment X is calculated as 1.09, and for treatment Y as 1.9. The overall difference between treatment X and Y is estimated at -0.82. Since the correlation of repeated mouse measurements is small, individual mouse effects have little influence on the calculation of expected gene expression values for groups. This means that the inclusion of mouse IDs as fixed or random effects do not have much practical influence in this particular example. For this reason, we observe similar results between this section and that of the previous section.

## Studies with covariates

### Background

In the remaining sections, we switch from looking at explanatory variables that are factors, and instead consider studies where the explanatory variable of interest is a covariate. Let us recall the basic models outlined in earlier sections, where a simple regression model for a covariate can be represented as a straight line defined by its y-intercept and slope. In the following section, we cover models that are more complex in their design, starting with a mix of covariates and factors. We also discuss options for non-linear fitted models that extend beyond the simple framework of y-intercept and slope. Whilst in practice the vast majority of study designs involve only factor variables, which we have covered extensively over multiple sections, this section is useful for the occasional study where the relationship between gene expression and a given covariate is of interest.

### Combination with factor variable

In the previous section we looked at models for the factors treatment and timepoint. We now consider a similar example, where there are two explanatory variables, the treatment factor and time as a covariate. The timepoints from the previous example is now treated as a numerical variable. Let us suppose that the timepoints T1 and T2 represent 1 hour post treatment and 2 hours post treatment, by either treatment X or Y. For simplicity, we do not consider having repeated mouse measurements here and assume that the measurements are taken from different mice each time. The data is as follows:



##    expression      id treatment time
## 1        1.01  MOUSE1         X    1
## 2        1.04  MOUSE2         X    1
## 3        1.04  MOUSE3         X    1
## 4        1.99  MOUSE4         X    2
## 5        2.36  MOUSE5         X    2
## 6        2.00  MOUSE6         X    2
## 7        2.89  MOUSE7         Y    1
## 8        3.12  MOUSE8         Y    1
## 9        2.98  MOUSE9         Y    1
## 10       5.00 MOUSE10         Y    2
## 11       4.92 MOUSE11         Y    2
## 12       4.78 MOUSE12         Y    2



**Figure 15.**
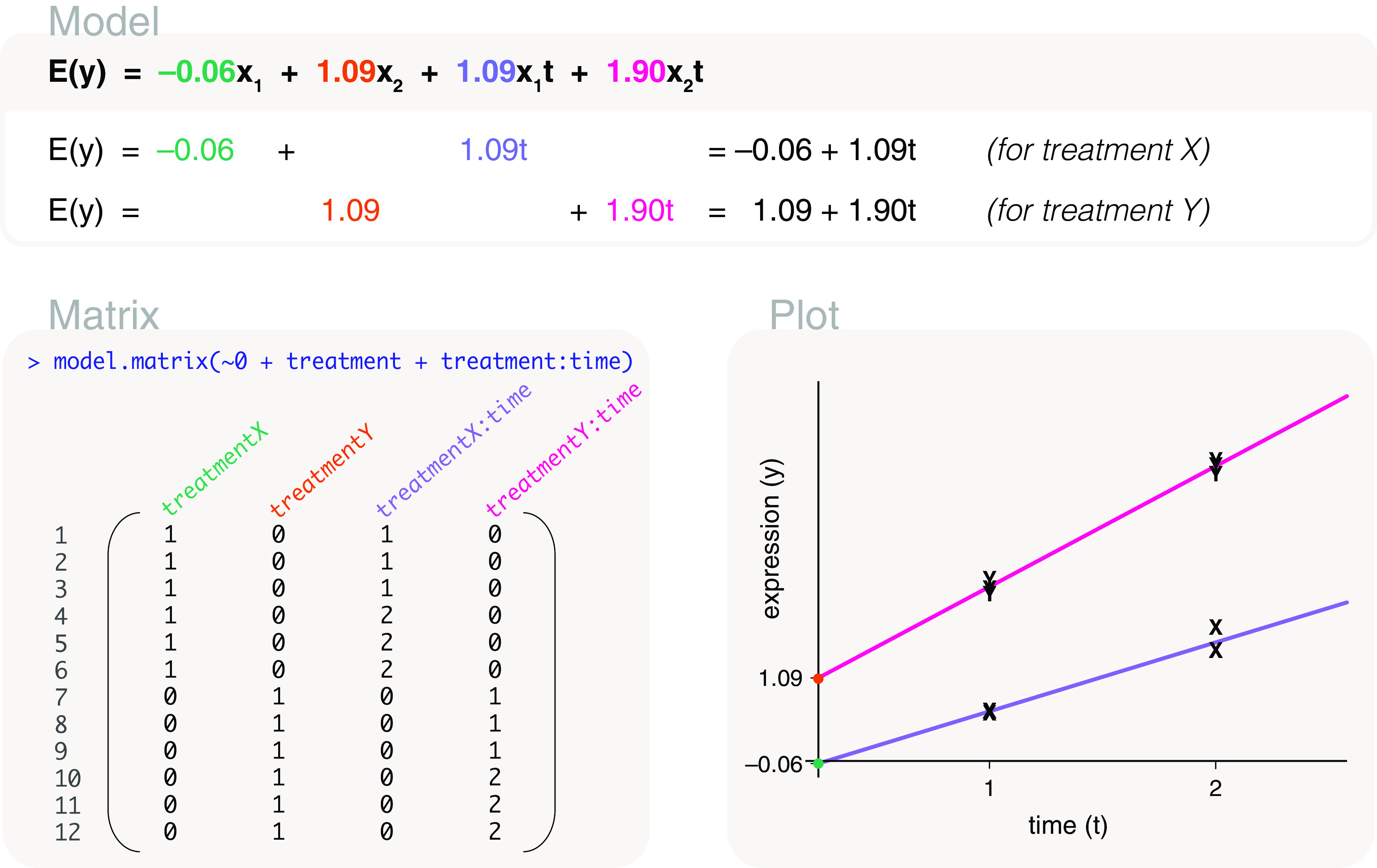
Expected gene expression is modelled by time as a covariate and treatment as a factor. In the plot, data points are labelled by treatment, and a fitted line is drawn for each of the treatments.

An appropriate design matrix that considers both the treatment factor and time covariate can be coded as model.matrix(~0+treatment+treatment:time), which gives a fitted line for each of the treatments ( Figure 15). For two fitted lines with the same slope, we could use the design matrix coded as model.matrix(~0+treatment+time). Let us recall that each regression line is defined by a y-intercept and slope. In our design matrix, the first and third columns are parameterised for the y-intercept and slope of the line modelling treatment X. The second and fourth columns are parameterised for the y-intercept and slope of treatment Y.

The y-intercepts for treatment X and treatment Y are estimated as -0.06 and 1.09 respectively. The y-intercepts, though, are generally not of interest. It is the slopes that are usually of interest since this quantifies the rate of increase or decrease in gene expression over time. The slope for treatment X is estimated as 1.09, and the slope for treatment Y is estimated as 1.9. In the previous section with the factors treatment and timepoint, time is consider as distinct changes in state from timepoint T1 to T2. Here, time as a covariate allows us to quantify the expected change in gene expression over an interval of time, such that over 0.5 units of time we can expect an increase of 0.54 in gene expression for treatment X (which is calculated by halving the third parameter estimate).

The fitted model can be written as *E*( *y*) = -0.06 *x*_1_ + 1.09 *x*_2_ + 1.09 *x*_1_ *t* + 1.9 *x*_2_ *t*, where the *x*_1_ and *x*_2_ are indicator variables for treatment X and treatment Y respectively, and *t* is a numerical variable representing time. Specifically, the model for treatment X can be written as *E*( *y*) = -0.06 + 1.09 *t*. The model for treatment Y can be written as *E*( *y*) = 1.09 + 1.9 *t*.

### Linear time series

We now consider a mouse study over several time points. This could represent a study examining gene expression changes in a developmental stage of interest, such as early embryonic development. There are gene expression measurements from mice at times 1, 2, 3, 4, 5 and 6, each in duplicate. The times could represent hours, days or weeks, with the data as follows:



##    expression   mouse time
## 1        2.08  MOUSE1    1
## 2        2.29  MOUSE2    1
## 3        3.58  MOUSE3    2
## 4        3.54  MOUSE4    2
## 5        3.66  MOUSE5    3
## 6        4.20  MOUSE6    3
## 7        2.56  MOUSE7    4
## 8        2.00  MOUSE8    4
## 9        0.81  MOUSE9    5
## 10       0.58 MOUSE10    5
## 11      -0.14 MOUSE11    6
## 12       0.14 MOUSE12    6



**Figure 16.**
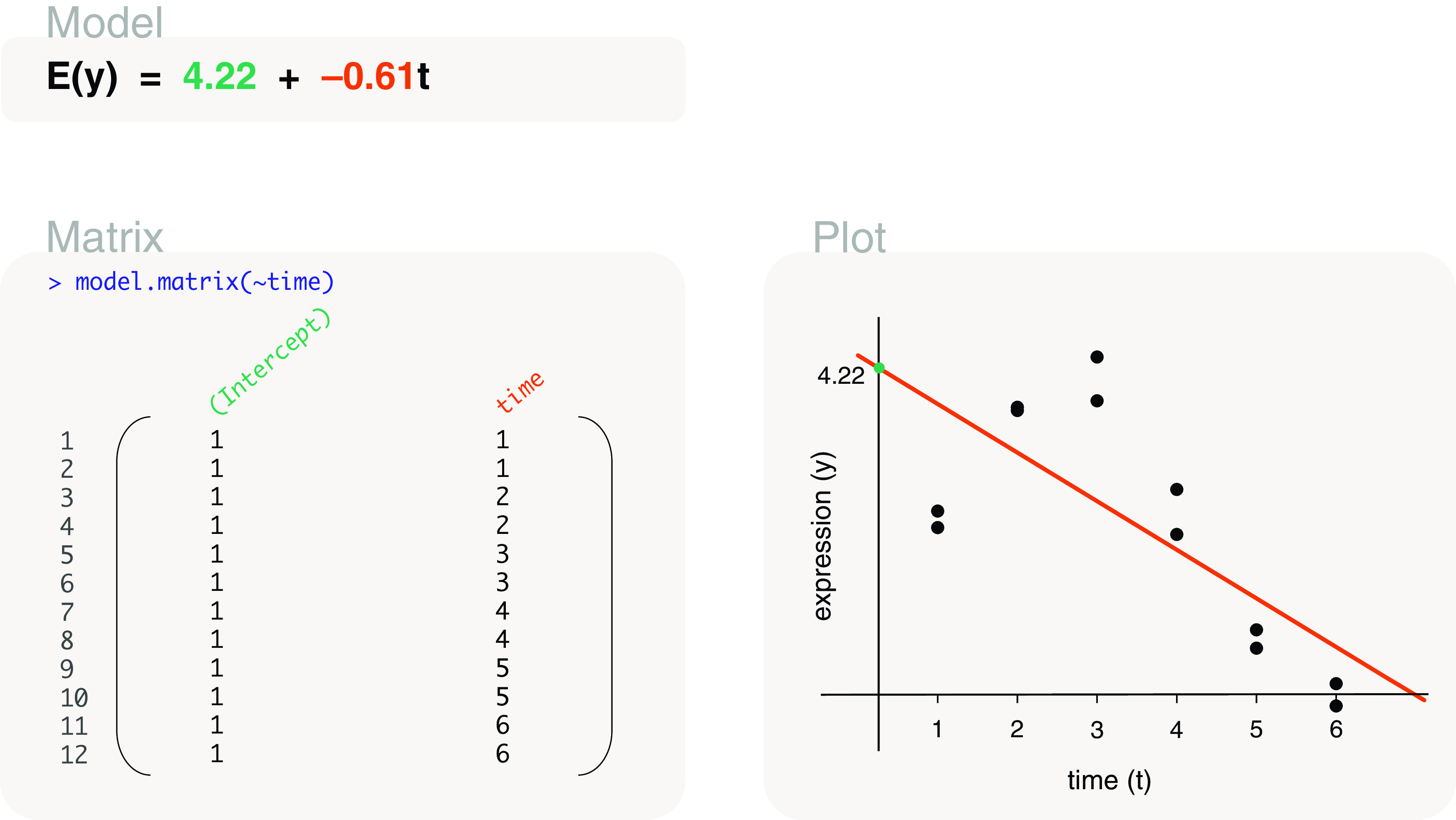
Expected gene expression is modelled by a time covariate, using a design matrix that includes an intercept term (linear fit).

Naturally, we use a design matrix coded as design <- model.matrix(~time) ( Figure 16). The design matrix contains two columns and is parameterised for the y-intercept (estimated here as 4.22) and the slope of the line (estimated as -0.6). Using this model, the gene expression is expected to decrease by 0.6 units for every unit increase in time.

### Quadratic time series

Looking more closely at the plot of expression versus time in Figure 16, we notice that expression seems to increase between time points 1 and 2, peaks between 2 and 3, and decreases between time points 3 and 6. So we also consider another model that describes the data using a curved line, such that the curved line takes the form of *y* = *a* + *bt* + *ct* ^2^ where *y* represents gene expression, *t* represents time and *t* ^2^ is calculated as the square of time (or time to the power of 2), and *a*, *b*, and *c* are model parameters which we estimate. Since the model includes the time covariate to the power of 2, the curve is referred to as a second degree polynomial or quadratic model.

**Figure 17.**
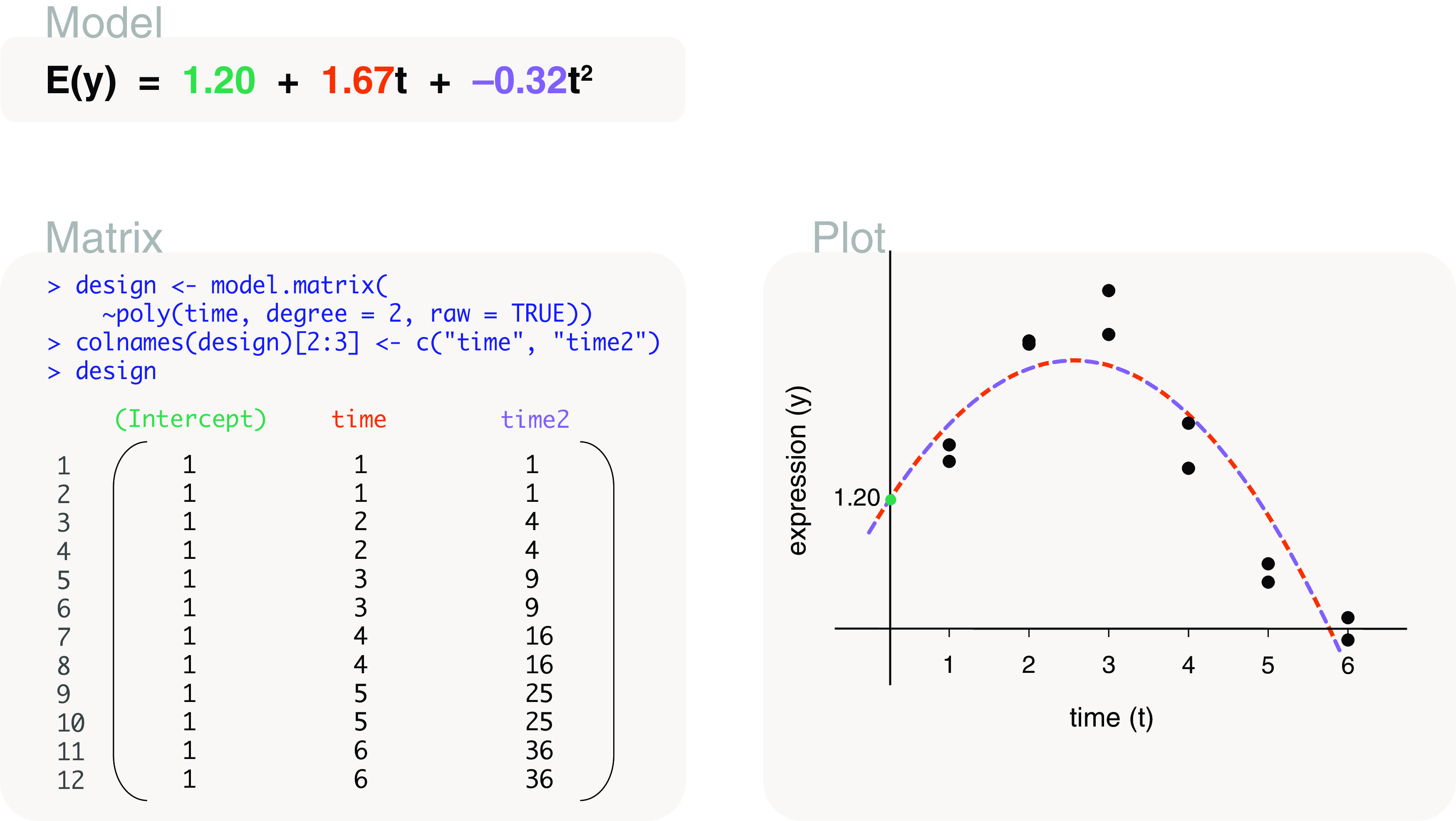
Expected gene expression is modelled by a time covariate, using a design matrix that fits a second degree polynomial (quadratic fit).

To fit such a model, the poly function from the **stats** package is used to specify the polynomial, with the specification that degree=2 for a second degree polynomial. The design matrix is coded as design <- model.matrix(~poly(time, degree=2, raw=TRUE)) (see Figure 17 where column names have been simplified). Using the design matrix, the parameters can be estimated as *a* = 1.2, *b* = 1.67, and *c* = -0.32. The fitted model can be written as *y* = 1.2 + 1.67 *t* + -0.32 *t* ^2^. The quadratic model has a the peak (or trough) occurring at *t* = − *b*/(2 *c*), which in our case is at time 2.57, and this translates to a maximum gene expression of 3.33. Using this model, we can say that gene expression is increasing up until time 2.57, and decreases after time 2.57. The quadratic model can help differentiate between the genes that are increasing in expression over time (where *b* > 0 and *c* = 0), genes that decreasing in expression over time ( *b* < 0 and *c* = 0), genes that increase in expression then decrease in expression ( *c* < 0), and genes that decrease then increase in expression ( *c* > 0).

In our example, we specify the use of raw polynomials via the raw argument in the poly function. This allows us to easily demonstrate what the linear component of the model ( *t* or time in the design matrix) and the quadratic component ( *t* ^2^ or time2 in the design matrix) looks like based on the data at hand. In practice, however, we would recommend using orthogonal polynomials rather than raw polynomials, by specifying raw=FALSE in the poly function. In fact, the default setting in poly computes orthogonal polynomials so the raw argument does not need to be specified. Raw polynomials can result in covariates that are correlated, which means that the importance of each individual effect is indistinguishable from each other. We can observe this correlation quite easily in our own example, by checking cor(poly(time, degree=2, raw=TRUE)). Orthogonal polynomials, on the other hand, ensure that the covariates are not correlated, which we can check with cor(poly(time, degree=2)), but give a messy demonstration of the parameters and model for the purpose of this article. Since the covariates are not correlated, orthogonal polynomials allows us to determine the genes where the linear term is important but the quadratic term is not, and vice versa. There can also be genes where both or neither linear and quadratic terms are important. This means that we recommend a design matrix coded as model.matrix(~poly(time, degree=2)) when fitting a quadratic model in practice, rather than the design matrix that is displayed in Figure 17. To compare the polynomial model against that of a constant term (i.e. gene expression does not change over time), an *F*-test can be used to test multiple parameters together (e.g. the linear and the quadratic term). In **limma**, we calculate *F*-statistics and their associated raw and adjusted *p*-values using the topTable function by specifying multiple columns in the coef argument, e.g. topTable(fit, coef=c(2,3), number=Inf) for such values across all genes as ranked by significance.

### Cubic time series

We extend our example with two additional time points, time 7 and 8, with the data as follows:



##    expression   mouse time
## 1        2.08  MOUSE1    1
## 2        2.29  MOUSE2    1
## 3        3.58  MOUSE3    2
## 4        3.54  MOUSE4    2
## 5        3.66  MOUSE5    3
## 6        4.20  MOUSE6    3
## 7        2.56  MOUSE7    4
## 8        2.00  MOUSE8    4
## 9        0.81  MOUSE9    5
## 10       0.58 MOUSE10    5
## 11      -0.14 MOUSE11    6
## 12       0.14 MOUSE12    6
## 13       1.13 MOUSE13    7
## 14       1.58 MOUSE14    7
## 15       3.45 MOUSE15    8
## 16       3.17 MOUSE16    8



**Figure 18.**
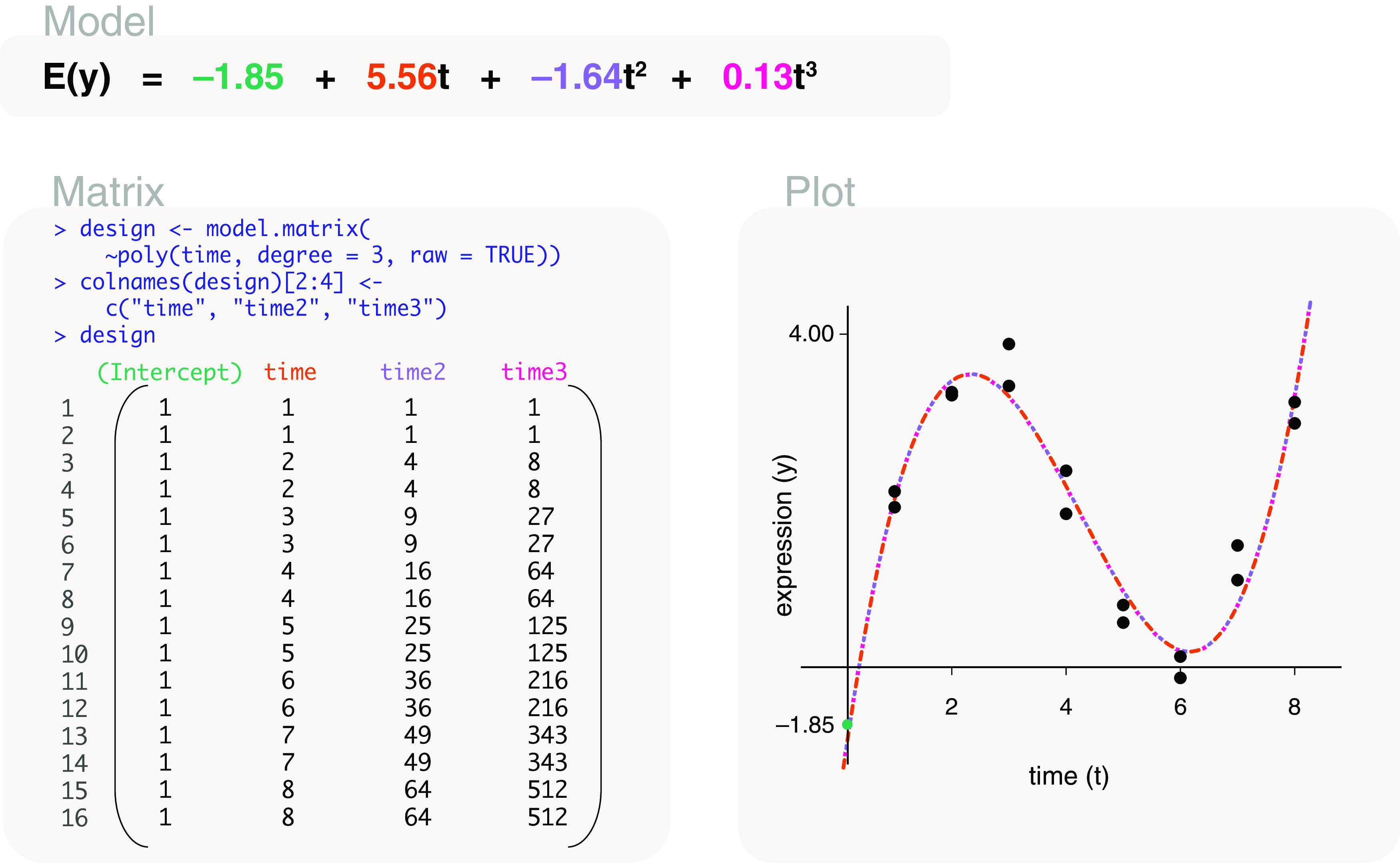
Expected gene expression is modelled by a time covariate, using a design matrix that fits a third degree polynomial (cubic fit).

We know from the previous section that between times 1 and 6, a linear fit to the data shows a decreasing trend ( Figure 16), and a quadratic fit to the same data shows an increasing trend before time 2.57 and a decreasing trend after time 2.57 ( Figure 17). The new data points, however, do not continue with the decreasing trend at time 7 and 8. For this reason, we fit a cubic model, or a third degree polynomial, to the data so that it can capture the two changes in gene expression trends. Like before, we use the poly function from the **stats** package to specify the polynomial we want to fit. The design matrix is coded as design <- model.matrix(~poly(time, degree=3, raw=TRUE)) (see Figure 18 where column names have been simplified). Note that we are again using a raw polynomial in this example for simplicity of illustration of the model parameters, although we would use an orthogonal polynomial in practice.

The four parameters in the model represent the intercept term, the coefficient for time (represented as *t*), the coefficient for time to the power of 2 (represented as *t* ^2^), and the coefficient for time to the power of 3 (represented as *t* ^3^). The third degree polynomial takes the form of *y* = *a* + *bt* + *ct* ^2^ + *dt* ^3^, where *y* represents gene expression and *t* represents time, and *a*, *b*, *c*, and *d* are model parameters which we estimate. The parameters can be estimated as *a* = -1.85, *b* = 5.56, *c* = -1.64, and *d* = 0.13. The fitted model can be written as *y* = -1.85 + 5.56 *t* + -1.64 *t* ^2^ + 0.13 *t* ^3^.

### Smooth curves

Other than polynomial models, another choice for fitting smooth curves to the data is via regression splines using the ns function for natural splines in the **splines** package. Examples of this can be found in the **limma** and **edgeR** user’s guides. In general, one would fit the most complex curve that one wishes to interpret and for which the number of time points can support. Usually the complexity of curve would never exceed that of the fifth order. For example, if there is no replication at time points, one might choose a second order polynomial ( degree=2) or a spline with 2 parameters ( df=2) for a study on 5 time points, this would leave 2 residual degrees of freedom for the model. If there are 10 time points, then a fifth degree polynomial or a spline with 5 parameters may be appropriate, resulting in a model with 4 residual degrees of freedom. Keep in mind that in gene expression analyses, the same model is applied to every gene in the dataset, even though a simpler model may be sufficient for some genes, whilst a more complex one is needed for others.

In the case where one wants to fit smooth curves to multiple groups where samples for the groups are taken at different time points, using regression splines allow the fitted trends to be compared between the groups whilst a polynomial fit does not. Furthermore, splines tend to give more sensible and stable curves with better behaviour at the boundaries of the fit than polynomials. On the other hand, polynomials such as the quadratic fit are handy for when one wants to determine the peak or trough of the fitted curve. Note that both the fitting of a spline or polynomial is particularly useful for time course experiments with lots of time points but no replication at each time point. The time series examples in the previous sections have replication at the time points. In that case, one could treat time as a factor rather than a covariate to avoid the interpretation of curves, and to obtain differences between distinct time points explicitly. This approach could be preferred by many.

### Cyclical models

We extend our example further to include an additional two time points, time 9 and 10. It turns out that the biology under study involves genes that are turned on and off cyclically over time. This example can represent studies on circadian rhythm where certain genes are turned on in the day and turned off at night, whilst others are on in the night and off in the day. The data is as follows:



##    expression   mouse time
## 1        2.08  MOUSE1    1
## 2        2.29  MOUSE2    1
## 3        3.58  MOUSE3    2
## 4        3.54  MOUSE4    2
## 5        3.66  MOUSE5    3
## 6        4.20  MOUSE6    3
## 7        2.56  MOUSE7    4
## 8        2.00  MOUSE8    4
## 9        0.81  MOUSE9    5
## 10       0.58 MOUSE10    5
## 11      -0.14 MOUSE11    6
## 12       0.14 MOUSE12    6
## 13       1.13 MOUSE13    7
## 14       1.58 MOUSE14    7
## 15       3.45 MOUSE15    8
## 16       3.17 MOUSE16    8
## 17       3.66 MOUSE17    9
## 18       4.08 MOUSE18    9
## 19       3.23 MOUSE19   10
## 20       2.93 MOUSE20   10



A cyclical model that models the rhythmic pattern in the data is considered for this example. Although the model itself may be complex, the interpretability of the model is greater than that of higher order polynomials since the fitted cyclical pattern is repeated over time. To fit a cyclical model, we use sin and cos functions from the **base** package to obtain the sine and cosine trigonometric functions on the time covariate. Now, let us consider an approximate cycle length. Our cycle length appears to be roughly 6 units - the first peak occurs at time 2.57 ( Figure 17), and the second peak occurs just after time 8 ( Figure 18). Note that a cycle length of 24 units would be appropriate for studies on circadian rhythm if time were measured in hours. The sine and cosine functions, with cycle length of 6 units, are coded as follows:



cycle <- 6
sinphase <- sin(2*pi*time/cycle)
cosphase <- cos(2*pi*time/cycle)



The design matrix is then coded as:



design <- model.matrix(~sinphase+cosphase)



**Figure 19.**
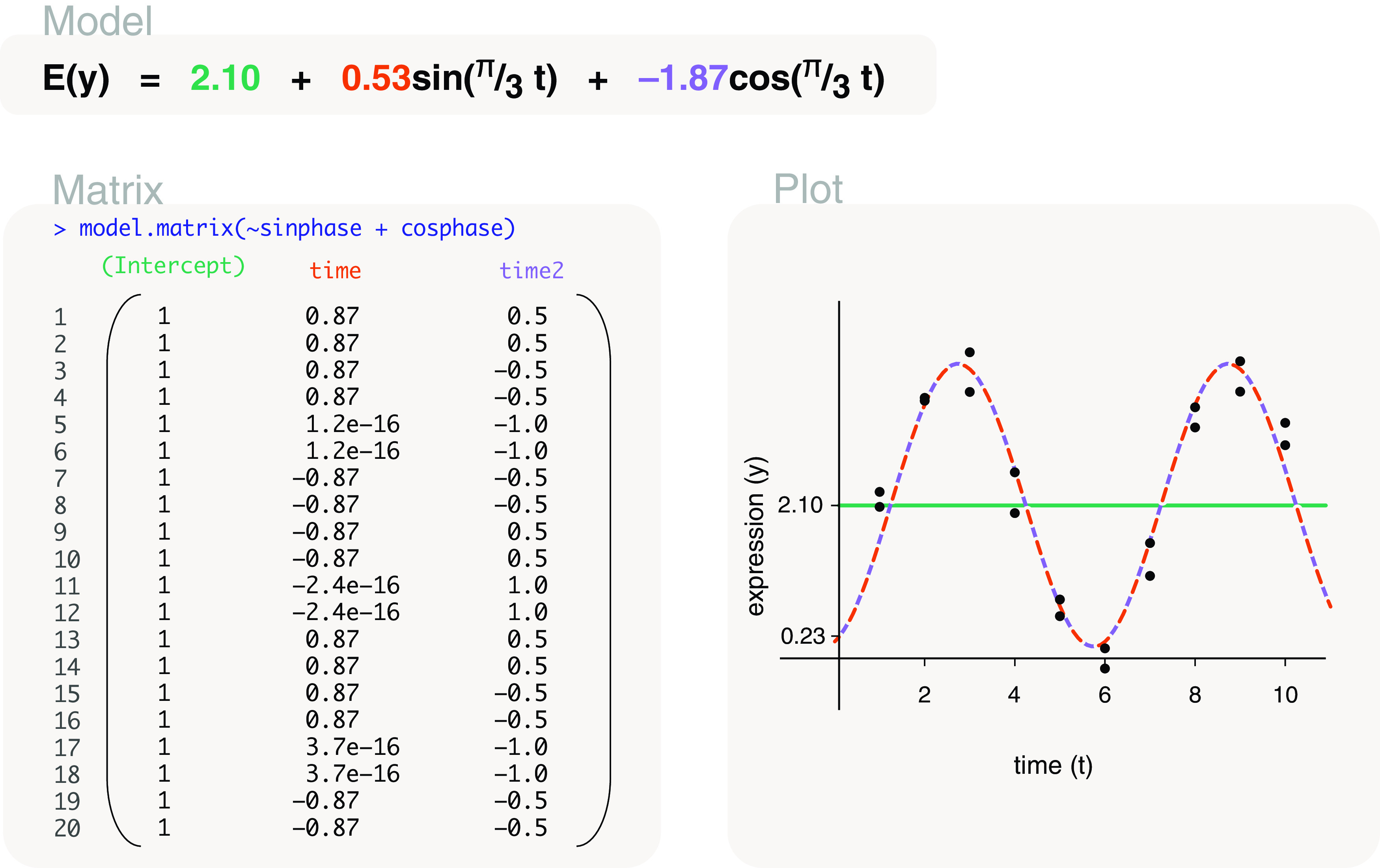
Expected gene expression is modelled using sin and cos functions (complete R code shown in the main article) to give a cyclical fit.

The first column in the design matrix ( Figure 19), or first parameter in the model, represents the horizontal shift of the cycling pattern from 0. The second column represents the amplitude (or height) of the sine function over time. Similarly, the third column represents the amplitude of the cosine function over time. The parameters can be estimated as 2.1, 0.53 and -1.87. The fitted model can be written as $E(y)=2.1+0.53\;\sin(\frac{\pi }{3}t)+-1.87\;\cos(\frac{\pi }{3}t)$, where *t* represents the time covariate.

**Figure 20.**
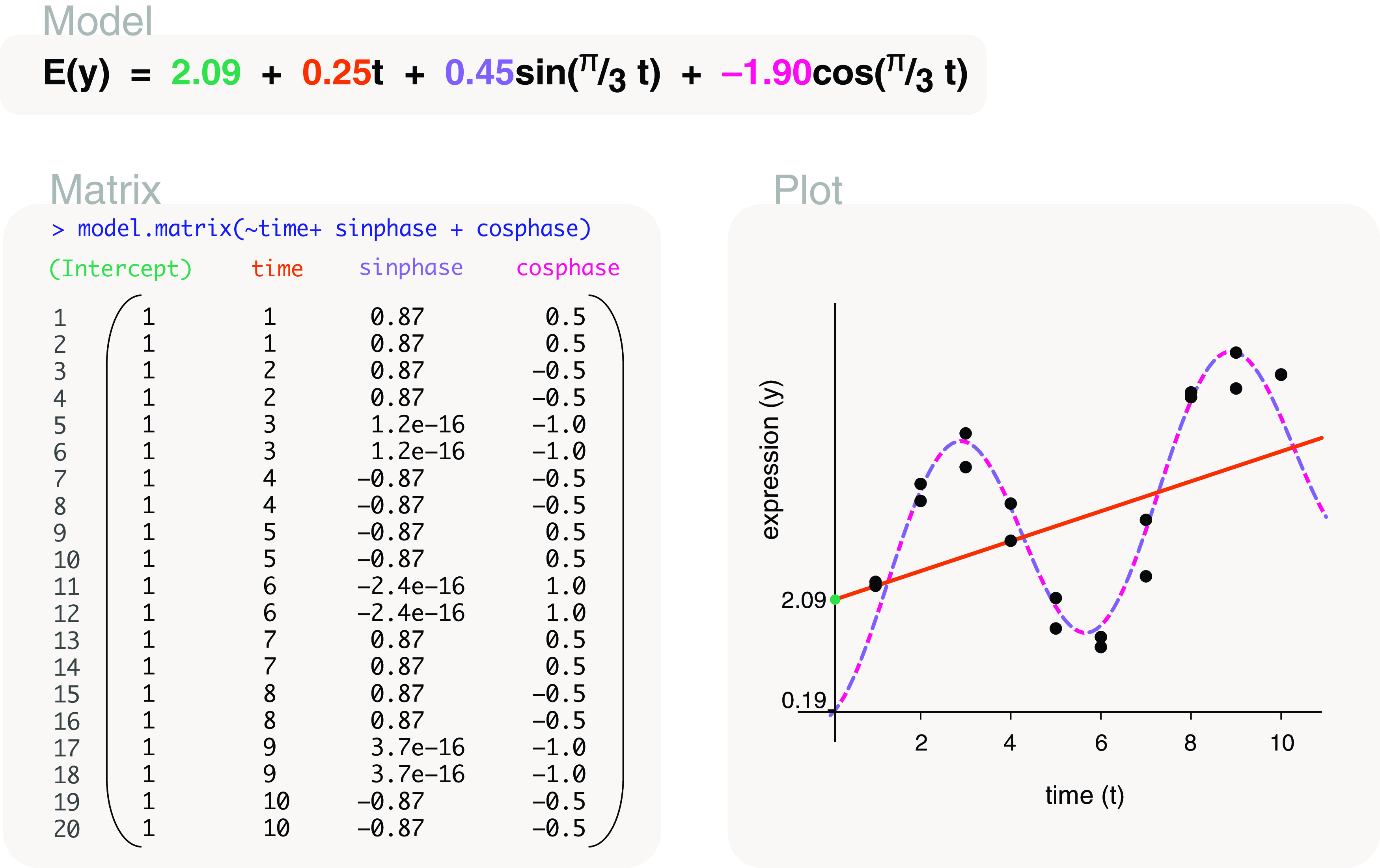
Expected gene expression is modelled using sin and cos functions (complete R code shown in the main article) to give a cyclical fit. An upwards trend in gene expression over time is also accounted for by including the time covariate in the design matrix.

The cyclical pattern can be integrated with other models in the data. If there is an upwards trend to the overall cyclical pattern in the data (data not shown), then we can include a linear component associated with time into the design matrix. The design matrix is coded as design <- model.matrix(~time + sinphase + cosphase), and the linear component is represented in the second column of the design matrix ( Figure 20). One can consider including a natural spline (using the ns function from the **splines** package) rather than a linear component, if the upward trend is more “curvy” and the linear trend is inadequate. For example, the natural spline can be included into the design matrix by coding as model.matrix(~ns(time, df=3) + sinphase + cosphase) (design matrix and model not shown).

## Further code notes

### Alternative code for design matrices

In this article, we have shown the coding of design matrices with an intercept term in the form of model.matrix(~variable) and those without an intercept term in the form of model.matrix(~0+variable) for an explanatory variable variable. There are other ways to code for the same design matrix, such as model.matrix(~1+variable) for a design matrix with an intercept term, and model.matrix(~-1+variable) or model.matrix(~variable-1) for a design matrix without an intercept term.

### Alternative code for contrast matrices

The makeContrasts function for creating contrast matrices ensures that the contrast matrix is ordered correctly according to model parameters in an associated design matrix. It also returns an error message if there is a mismatch with the associated design matrix which is helpful. However, the makeContrast functions requires one to type full column names from the design matrix which can be tedious. The function also complains (in the form of a warning message) about column names from the design matrix that are syntactically invalid.

Alternatively, one can create a contrast matrix manually by using the cbind function as follows:



contrasts <- cbind(
  AvsC=c(1,0,-1,0), BvsC=c(0,1,-1,0), ABvsCD=c(0.5,0.5,-0.5,-0.5))
rownames(contrasts) <- LETTERS[1:4]
contrasts

##   AvsC BvsC ABvsCD
## A    1    0    0.5
## B    0    1    0.5
## C   -1   -1   -0.5
## D    0    0   -0.5



The above contrast matrix contains three contrasts, which are linear combinations of four model parameters (A, B, C and D). The first contrast compares A to C, the second compares B to C, and the last contrast compares the average of A and B to the average of C and D. When coding for contrast matrices manually, one should carefully check that the rows in the contrast matrix match the columns of the design matrix.

### Example code for a *limma* workflow

Starting with a counts table, a complete workflow for differential gene expression analysis of RNA-seq data using the **limma** package can be found in the “ *RNA-seq analysis is easy as 1-2-3 with limma, Glimma and edgeR*” workflow article ^5^. A summary of the main steps for fitting a linear model to each gene and obtaining parameter estimates are as follows:



group <- as.factor(c(1,1,1,2,2,2))
design <- model.matrix(~0+group)
contrasts <- makeContrasts(group1-group2, levels=colnames(design)) 
v <- voom(counts, design)
fit <- lmFit(v, design)
fit <- contrasts.fit(fit, contrasts)
fit <- eBayes(fit)
topTable(fit)



The above code is non-runnable as the counts object is missing. The counts object here is assumed to be a table of counts, with rows as genes and columns as samples. In this example, there are six samples, three of which belong to group 1 and the other three to group 2. The design matrix is parameterised for a means model, and the contrast matrix is used to calculate the difference in mean expression between group 1 and group 2.

## Theory of linear regression models, design matrices and contrast matrices

This section briefly summarises the mathematics of design and contrasts matrices. This section is optional and understanding it is not required to undertake any of the analyses described earlier in the article. It is provided as a reference for those comfortable with the mathematical notation.

A regression model, in the general sense, can be used to describe the relationship between explanatory variables and gene expression, the response variable. There are many different types of regression models, where each assume different characteristics for the relationship between explanatory and response variables, as well as the properties associated with variability in the data. Consider one such model, a simple linear regression model

$$E(y)=\beta_{0}+\beta_{1}x$$

which describes the expected value for gene expression *E*( *y*) as equal to the sum of a constant *β*_0_ and an explanatory variable *x* scaled by a coefficient *β*_1_. The *β* values ( *β*_0_ and *β*_1_) are referred to as regression parameters, where their real values are unknown. In matrix notation, the right-hand-side of the equation, *β*_0_ + *β*_1_ *x*, can be written as

$$X\beta =\left[\begin{array}{cc} 1 & x \end{array}\right]\left[\begin{array}{c} \beta_{0}\\ \beta_{1} \end{array}\right]$$

where it is broken down into the matrix **X** and the regression parameters ***β***. The values along the row in **X** are multiplied by the regression parameters, such that it calculates *β*_0_ × 1 + *β*_1_ × *x*. Since regression parameters are unknown, they are estimated from a study of *n* samples. For a given gene, gene expression values are denoted as **y** = *y*_1_, *y*_2_, *y*_3_,..., *y*_*n*_ for the *n* samples. The matrix **X**, which we refer to as the design matrix, is used to store values of the explanatory variable associated with each sample, such that

$$X=\left[\begin{array}{cc} 1 & x_{1}\\ 1 & x_{2}\\ 1 & x_{3}\\ 1 & .\\ 1 & .\\ 1 & x_{n} \end{array}\right]$$

with each row representing a sample. Putting the response and explanatory variables together, we can then solve the equation

y=Xβ+e. (1)

to obtain estimates for parameters ***β***. The **e** denotes differences between observed gene expression values and the true population value (e.g. the population mean), where **e** is referred to as the errors. The above equation ( Equation 1) can be expanded out and written as

$$\left[\begin{array}{c} y_{1}\\ y_{2}\\ y_{3}\\ .\\ .\\ y_{n} \end{array}\right]=\left[\begin{array}{cc} 1 & x_{1}\\ 1 & x_{2}\\ 1 & x_{3}\\ 1 & .\\ 1 & .\\ 1 & x_{n} \end{array}\right]\left[\begin{array}{c} \beta_{0}\\ \beta_{1} \end{array}\right]+\left[\begin{array}{c} e_{1}\\ e_{2}\\ e_{3}\\ .\\ .\\ e_{n} \end{array}\right].$$

Through an estimation process, we obtain parameter estimates which we denote as $\hat{\beta }$. Using the parameter estimates, we can calculate fitted values for gene expression by multiplying the design matrix **X** by the parameter estimates, such that the fitted values are calculated as $\hat{y}=X\hat{\beta }$. The least squares estimation strategy obtains estimates of regression parameters by minimising the sum of squared residuals, where residuals are calculated as the difference between observed **y** and fitted gene expression values $\hat{y}$.

The simple linear regression model, *E*( *y*) = *β*_0_ + *β*_1_ *x*, can be generalised and extended to contain more explanatory variables and written as

$$E(y)=\beta_{1}x_{1}+\beta_{2}x_{2}+\beta_{3}x_{3}+...+\beta_{p}x_{p}.$$

We refer to this type of model as a linear regression model. The model contains *p* regression parameters, each of which are associated with an explanatory variable. In a study on *n* samples, an associated design matrix **X** can be written as

$$X=\left[\begin{array}{ccccc} x_{1,1} & x_{2,1} & x_{3,1} & ... & x_{p,1}\\ x_{1,2} & x_{2,2} & x_{3,2} & ... & x_{p,2}\\ x_{1,3} & x_{2,3} & x_{3,3} & ... & x_{p,3}\\ . & . & . & ... & .\\ . & . & . & ... & .\\ x_{1,n} & x_{2,n} & x_{3,n} & ... & x_{p,n} \end{array}\right],$$

where the values *x*_*i*, *j*_ represent explanatory variable *i* in sample *j*, for *i* = 1,2,3,...., *p* and *j* = 1,2,3,..., *n*. A model with an intercept term, simply sets the values in the first column of the design matrix as 1. To distinguish between a model with and without an intercept term, the associated parameter for the intercept term is sometimes denoted as *β*_0_ rather than *β*_1_, although either notation is acceptable.

Contrasts are set up to examine relationships of interest, such that a contrast matrix **C** contains *K* column vectors of length *p* (number of model parameters), and can be written as

$$C=\left[\begin{array}{ccccc} c_{1,1} & c_{2,1} & c_{3,1} & ... & c_{K,1}\\ c_{1,2} & c_{2,2} & c_{3,2} & ... & c_{K,2}\\ c_{1,3} & c_{2,3} & c_{3,3} & ... & c_{K,3}\\ . & . & . & ... & .\\ . & . & . & ... & .\\ c_{1,p} & c_{2,p} & c_{3,p} & ... & c_{K,p} \end{array}\right].$$

Each column in **C**, or individual contrast **c**_*k*_ = *c*_*k*,1_, *c*_*k*,2_, *c*_*k*,3_,...., *c*_*k*, *p*_, represents a relationship or comparison of interest, where for each gene we test whether or not *δ*_*k*_ = **c**_*k*_^*T*^ ***β*** is non-zero. *δ*_*k*_ can be expanded out and written as

$$\delta_{k}=\left[\begin{array}{ccccc} c_{k,1} & c_{k,2} & c_{k,3} & ... & c_{k,p} \end{array}\right]\left[\begin{array}{c} \beta_{1}\\ \beta_{2}\\ \beta_{3}\\ ...\\ \beta_{p} \end{array}\right].$$

## Discussion

In this article, we described the set up and interpretation of design matrices and their associated models for a variety of case studies involving factors and/or covariates. The examples in this article are completely reproducible via our Rmarkdown document that can be downloaded from the **RNAseq123** workflow package available from https://doi.org/doi:10.18129/B9.bioc.RNAseq123. The document can be used to recreate design matrices and plots found in this article, as well as to obtain estimated values for model parameters.

The estimation process was not described explicitly in our work since it is not of direct interest here. Parameter estimates were merely used to illustrate aspects of the model relating to the design matrix. For simplicity, parameter estimation in our single gene examples was carried out using the lm function from the **stats** package, with the exception of the mixed effects model that uses lmFit from **limma** due to its complexity. In practice, **limma**’s lmFit function would be used to obtain parameter estimates for multiple genes simultaneously in RNA-seq datasets and other genomic data types. The estimation process performed by lm and lmFit can be different ( lm carries out ordinary least squares estimation, whereas lmFit usually carries out weighted least squares estimation), so their parameter estimates may differ also. The two functions would produce the same parameter estimates if lmFit was run in its simplest form without intrablock correlations, precision weights or robustification.

Our article describes case studies that are common to the field of genomics research, where the choice of language used throughout the article makes it easily adaptable to studies and datasets for various applications. We have taken special care to explain, where appropriate, the reasoning behind specific design matrix set ups and describe how one would go about interpreting the associated model and its parameters. This allows readers to build their knowledge and understanding of simpler concepts, and work their way up to more advanced concepts, such as mixed effects or cyclical models, that are also described. Although we have covered design matrices in many common experimental settings, there will certainly be more complex scenarios that have been missed. We do not describe, for example, a study with a covariate and multiple factors, however, a reader should be able to tackle such an example quite easily with their understanding from the sections on multiple factors, combined with their understanding from the studies on covariates. For more complicated experimental designs, we would advise readers to consult with a statistician or bioinformatician who is experienced in linear modelling.

## Software availability

This article was written using Bioconductor ^10^ version 3.12, running on R version 4.0.3 (2020-10-10). The examples in this article made use of the software packages **limma** version 3.45.19 and **TeachingDemos** ^11^ version 2.12. This article was written as an Rmarkdown document that was compiled using knitr, and converted from an Rmarkdown document to LaTex with the help of **BiocWorkflowTools** version 1.15.0. All packages and their version numbers are shown below. Reproducible code for this article is available at https://doi.org/doi:10.18129/B9.bioc.RNAseq123 (Artistic License 2.0), which also stores the code for the ” *RNA-seq analysis is easy as 1-2-3 with limma, Glimma and edgeR*” workflow article ^5^.



sessionInfo()

## R version 4.0.3 (2020-10-10)
## Platform: x86_64-apple-darwin17.0 (64-bit)
## Running under: macOS Mojave 10.14.6
##
## Matrix products: default
## BLAS:   /System/Library/Frameworks/Accelerate.framework/Versions/A/Frameworks
    /vecLib.framework/Versions/A/libBLAS.dylib
## LAPACK: /Library/Frameworks/R.framework/Versions/4.0/Resources/lib/libRlapack.dylib
##
## locale:
## [1] en_AU.UTF-8/en_AU.UTF-8/en_AU.UTF-8/C/en_AU.UTF-8/en_AU.UTF-8
##
## attached base packages:
## [1] stats     graphics  grDevices utils     datasets  methods   base
##
## other attached packages:
## [1] TeachingDemos_2.12 limma_3.45.19
##
## loaded via a namespace (and not attached):
##  [1] rstudioapi_0.11          knitr_1.30               magrittr_1.5
##  [4] usethis_1.6.3            BiocWorkflowTools_1.15.0 statmod_1.4.35
##  [7] here_0.1                 R6_2.4.1                 rlang_0.4.8
## [10] stringr_1.4.0            httr_1.4.2               tools_4.0.3
## [13] xfun_0.18                git2r_0.27.1             htmltools_0.5.0
## [16] yaml_2.2.1               digest_0.6.27            rprojroot_1.3-2
## [19] bookdown_0.21            BiocManager_1.30.10      fs_1.5.0
## [22] glue_1.4.2               evaluate_0.14            rmarkdown_2.5
## [25] stringi_1.5.3            compiler_4.0.3           backports_1.1.10


