# Supplementary material for: A guide to creating design matrices for gene expression experiments
Source: F1000Res. 2020 Dec 10;9:1444. [Version 1] doi: 10.12688/f1000research.27893.1 (PMC7873980; doi:10.12688/f1000research.27893.1)
Supplement: Peer review history

# Peer review: Reviewer response for version 1

**DOI:** 10.5256/f1000research.30844.r76092

**Published:** 2021-02-09

**Authors:** Jenny Drnevich

**Affiliations:** 1 High-Performance Biological Computing Group, University of Illinois, Urbana-Champaign, IL, USA

Law *et al.*’s Method Article focuses on two crucial aspects of differential expression analysis: how to properly form the statistical model and how to pull appropriate contrasts from the model. Unlike other aspects involved with a full analysis that are generalizable to all experiments (e.g., sample quality control, normalization, filtering, visualization of results and down-stream data mining), the statistical modeling must be tailored for each experimental design and hence why it often is the most difficult part for beginners and experienced analysts alike. Technical mathematical notation is confined to one “optional” section, so the rest of the manuscript is more understandable to people from varied backgrounds that are looking for help with making correct design and contrast matrices. Although the manuscript is more understandable, it still contains such necessary depth of information spanning simple to very complex models that it will require many readings to fully digest all of it. I envision this to become a welcome guide to be referenced again and again.

 This manuscript distinguishes itself from other guides on selecting design and contrast matrices like the limmaUsersGuide() and edgeRUsersGuide(), which are excellent but also contain a tome of information on other aspects of gene expression analysis such that the design/contrast matrix is not as easy to find as this stand-alone article. I also really like the combination in each figure of the fitted model statements (e.g., E(y) = 1.85 + 0.54x), the actual design matrix and a plot visualizing the data points and estimated parameter values. The fitted model statements are an addition that I have not seen much in other references on making design matrices. I do have a few minor points below that should be addressed:

1. Why are all the makeContrasts() functions called with levels=colnames(design) instead of just levels=design? It seems the second way is not only simpler but also reinforces the idea that the contrast matrix must be defined from the design matrix. I am not sure I have seen the first way in any other documentation in making contrast matrices, so it seems like an explicit change but without a compelling rationale.
2. At the end of the “Contrast matrix for computing differences “ section, the sentence “gene expression of healthy mice is greater than sick mice by a value of −1.62” required several readings to understand what it was trying to say, probably because of the conflict between “greater than” and “-1.62”. I would suggest replacing “greater than” in this sentence and the one above with “different than”.
3. In the “2 versus 2 group comparisons” section, I question the choice to compare control and treatment III against treatment I and II given the rationale “This may be of interest if there are prior expectations that two groups are more similar to each other than the other two”. Given that we have already seen that treatment III has the biggest change from the control, I feel like readers will be confused as to why control and treatment III were paired together instead of control and treatment I.
4. I am somewhat disappointed not to see the “sum to zero” parametrization for a two-factor model that is in the limmaUsersGuide(). Upon reflection, I agree with the decision to not add yet another model type to this already complex guide which does contain example contrasts to get the traditional main effects contrasts.
5. Would unequal sample sizes in groups affect the results of the modeling in any way? Perhaps this only applies to the estimate of the grand mean in the “sum to zero” parametrization, but I have occasionally struggled with getting the same answers from different model parametrizations and on-line help has suggested it was due to uneven sample sizes in the groups. If uneven sample sizes would not affect the choice of parametrization or formation of contrasts, it would be nice to have a simple sentence of that somewhere. If there are situations where uneven sample sizes would be an issue but it is beyond the scope of this paper, that should be addressed briefly and outside references given.

Is the rationale for developing the new method (or application) clearly explained?

Yes

Is the description of the method technically sound?

Yes

Are the conclusions about the method and its performance adequately supported by the findings presented in the article?

Yes

If any results are presented, are all the source data underlying the results available to ensure full reproducibility?

Yes

Are sufficient details provided to allow replication of the method development and its use by others?

Yes

Reviewer Expertise:

Gene expression analysis, bioinformatics, RNA-Seq data,

I confirm that I have read this submission and believe that I have an appropriate level of expertise to confirm that it is of an acceptable scientific standard.

# Peer review: Reviewer response for version 1

**DOI:** 10.5256/f1000research.30844.r76094

**Published:** 2021-02-05

**Authors:** Federico Marini

**Affiliations:** 1 Institute of Medical Biostatistics, Epidemiology and Informatics, University Medical Center of the Johannes Gutenberg University Mainz, Mainz, Germany

This manuscript presents a detailed description of an essential aspect of the differential expression analysis workflows, applicable to a variety of high-throughput assays, namely the creation, specification, and comprehension of proper design matrices, followed by the correct setup for the relevant comparisons, to be done via contrast matrices.

 While technologies such as RNA-seq are widespread in the current literature, and an extensive collection of software packages for such datasets are nowadays well established, the appropriate choices in statistical modeling might remain a more elusive topic to many researchers with experience in genomic data analysis, but not formally trained in biostatistics/bioinformatics.

 Seeing over the years the amount of questions on specialized support forums (the Bioconductor Support Site, Stack Overflow, Biostars) about the topics addressed in this work, I can only say it is a very welcome and valuable contribution to the scientific literature, and additionally become an essential resource to be used for teaching (e.g. in lectures but also in more focused workshops, as it is written in a way that it integrates seamlessly with the widely used framework of limma).

 Overall, the manuscript is clearly written, avoiding overcomplicated jargon to make it understandable to a large readership, and well structured in a way that builds up from the fundamental elements of commonly encountered experimental designs, followed by more complex yet plausible scenarios, where more refined approaches might be required to properly address the underlying scientific questions.

 I have a few points that a revised version of the manuscript might address to improve the overall quality of this work:

- The text could refer to Figure 1 subpanels explicitly, for more clarity.
- Moreover, in the center and right panel the description and legend both refer to Beta1 and Beta2, while the figure shows Beta0 and Beta1.
- Some formal definitions in the Terminology table can be improved with more complete wordings to provide the clearest, non-ambiguous, yet formally correct descriptions of the fundamental concepts.
- Response variable: add something like "often specified/referred to as Y in this manuscript".
- Explanatory variable: add "often noted as X_i" or similar.
- Nested factors: does this definition need e.g. the addition of "mutually exclusively" for better clarity?
- Mixed effect models: "where random effects are usually not of interest to the study at hand.", probably add something like "yet need to be specified for proper modelling of the data at hand"? Related to this, a similar formulation could be adopted at page 19 when defining the random effect as "not of interest to the study" - while this is truly not of interest, the omission of accounting for that could be detrimental in the modeling.
  Overall, I think that if the definitions in here would be a little more complete, it would increase a lot the value of this table as a go-to reference for many occasions - of course, without overcomplicating too much by overloading it with theoretical details.
- As a general comment - for the table and the main text: I am not so sure that the "covariate" refers only to the continuous variables; I often encountered the nomenclature of a "categorical covariate". I suggest it is worth revisiting this aspect and if needed clarify it further in the scope of this manuscript.
- In the subsection "Studies with covariates", the cyclical models are not included in the table just below the text.
- It could be helpful to have a visual representation for the "Overlap of treatment-control results" with something like a Venn Diagram or an Upset plot to provide an immediate summary of the textual description.
- While I understand that it could be out of scope for this manuscript, focused on the limma framework, it might be worth mentioning that some conventions might differ in other frameworks. For example, DESeq2 specifies the main variable of interest as the last term in the design, while on page 22 it is stated "This is why we place the factor of interest first as it simplifies the subsequent code for the comparisons of interest, even though a different order of factors added give equivalent models with variations in parameterisation.". The authors could mention that different software might follow different default implementation and recommend to accurately read the software documentation.
- On page 36, the term delta_k was not previously defined (but was previously used for the interaction term). Does this need to be clarified explicitly?
- It might concern the pagination of the article, but sometimes it is not entirely clear where the output of the code is close to a figure which actually relates to a section above (e.g. on page 10 with Figure 4). Another example where the sequence of text-output-figures is not so clear is on page 33 (Model belongs to above section, Matrix + Plot to the one below).
- The authors could provide a snapshot of the RNAseq123 repository (e.g. on Zenodo) to guarantee the complete reproducibility of the manuscript - often times vignettes are edited over the development cycles. Even if not entirely a "Software" article, it is inserted into a workflow software package in Bioconductor.

Some sentences are missing some prepositions or are not fully correct from the grammatical point of view. I am reporting here some other sentences as well that would need to be fixed:

-  Abstract: "set up *of* an appropriate model via design matrices", "Differential expression analysis [...] use*s* linear models".
-  page 4, "where the line is defined by Beta0 the y-intercept and Beta1 the slope" should have some commas inserted.
-  "classifiers associated with samples", the wording is not so clear.
-  page 8, "if variable is a covariate the then", invert the and then.
-  Legend of Figure 3, the backticks for rendering the code monospace font are still visible.
-  page 12: refers to the `makeContrast` function, but should read `makeContrasts` (also page 17, please check all occurrences).
-  page 23: "the use of unsupervised clustering plots" could be rephrased in a more general way as "the use of exploratory data analysis techniques". In the sentences after that: "with one addition*al* factor to observe", please correct this.
-  page 28: "time is consider*ed* as distinct changes in state".
-  page 35: Section "Theory of linear regression models, design matrices and contrast matrices" might be missing an oxford comma.
-  page 36, "A model with an intercept term, simply sets", please remove the comma.
-  page 37, "R markdown" should read "R Markdown" (at least it does so in most of RStudio's documentation)

Most of these points are easily addressable, and I believe they would contribute in making even clearer the concepts already nicely described throughout this work, which I envision to be extremely useful for many practitioners that have to deal with (and properly perform) omics data analysis.

Is the rationale for developing the new method (or application) clearly explained?

Yes

Is the description of the method technically sound?

Yes

Are the conclusions about the method and its performance adequately supported by the findings presented in the article?

Yes

If any results are presented, are all the source data underlying the results available to ensure full reproducibility?

Yes

Are sufficient details provided to allow replication of the method development and its use by others?

Yes

Reviewer Expertise:

Bioinformatics, RNA-seq data analysis

I confirm that I have read this submission and believe that I have an appropriate level of expertise to confirm that it is of an acceptable scientific standard.
